# Ancient Anxiety Pathways Influence *Drosophila* Defense Behaviors

**DOI:** 10.1016/j.cub.2016.02.031

**Published:** 2016-04-04

**Authors:** Farhan Mohammad, Sameer Aryal, Joses Ho, James Charles Stewart, Nurul Ayuni Norman, Teng Li Tan, Agnese Eisaka, Adam Claridge-Chang

**Affiliations:** 1 Program in Neuroscience and Behavioral Disorders, Duke-NUS Graduate Medical School, Singapore 138673, Singapore; 2 Institute for Molecular and Cell Biology, Agency for Science Technology and Research, Singapore 138673, Singapore; 3 Department of Physiology, National University of Singapore, Singapore 138673, Singapore

## Abstract

Anxiety helps us anticipate and assess potential danger in ambiguous situations [1, 2, 3]; however, the anxiety disorders are the most prevalent class of psychiatric illness [4, 5, 6]. Emotional states are shared between humans and other animals [7], as observed by behavioral manifestations [8], physiological responses [9], and gene conservation [10]. Anxiety research makes wide use of three rodent behavioral assays—elevated plus maze, open field, and light/dark box—that present a choice between sheltered and exposed regions [11]. Exposure avoidance in anxiety-related defense behaviors was confirmed to be a correlate of rodent anxiety by treatment with known anxiety-altering agents [12, 13, 14] and is now used to characterize anxiety systems. Modeling anxiety with a small neurogenetic animal would further aid the elucidation of its neuronal and molecular bases. *Drosophila* neurogenetics research has elucidated the mechanisms of fundamental behaviors and implicated genes that are often orthologous across species. In an enclosed arena, flies stay close to the walls during spontaneous locomotion [15, 16], a behavior proposed to be related to anxiety [17]. We tested this hypothesis with manipulations of the GABA receptor, serotonin signaling, and stress. The effects of these interventions were strikingly concordant with rodent anxiety, verifying that these behaviors report on an anxiety-like state. Application of this method was able to identify several new fly anxiety genes. The presence of conserved neurogenetic pathways in the insect brain identifies *Drosophila* as an attractive genetic model for the study of anxiety and anxiety-related disorders, complementing existing rodent systems.

## Highlights

• *Drosophila* orthologs of anxiety genes affect fly wall following
• Conserved anxiety genes influence fly defense behaviors similarly to mouse anxiety
• New candidate anxiety genes are identified from fly defense behavior screen
• *Drosophila* identified as a new neurogenetic tool for anxiety research

## Graphical Abstract

**Figure:**
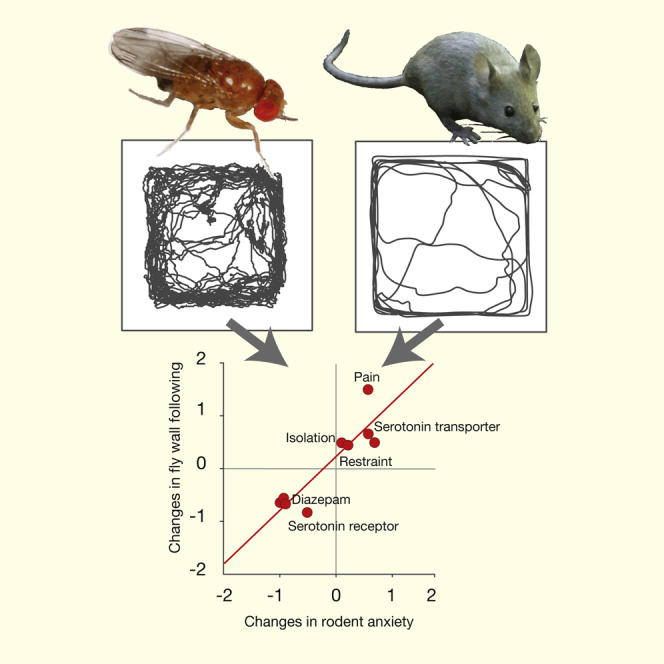

## Teaser

Mohammad et al. show that orthologs of mammalian anxiety factors govern defense behaviors in the fly and use these behaviors to identify new conserved candidate anxiety genes. Thus, rodent anxiety research may be complemented by *Drosophila* neurogenetic models.

## Results

### Flies Follow the Walls of an Enclosed Chamber

**Figure 1:**
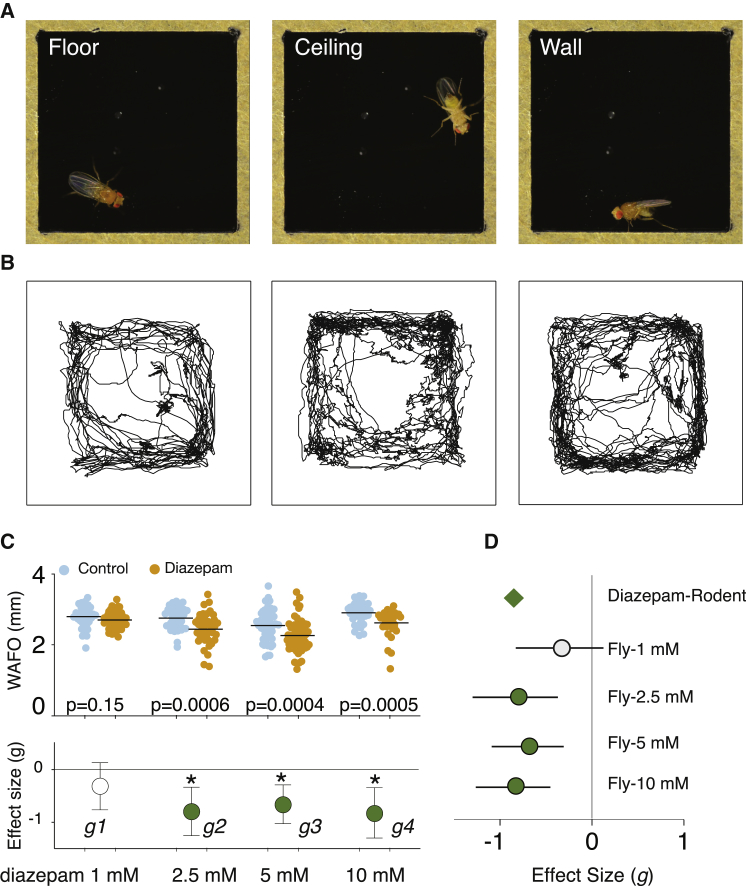
*Drosophila* Wall Following Behavior Is Reduced by Diazepam (A) Flies in a glass-topped arena walk on all interior surfaces. (B) Tracking data from a 10-min experiment reveal that flies mainly walk in the perimeter of the arena. (C) Flies fed with diazepam had decreased WAFO compared with controls (*g1* = −0.32, *g2* = −8.0, *g3* = −0.67, *g4* = −0.83, n = 40, 40). Fly WAFO was measured as mean distance from center in millimeters. Dots indicate the mean distance from center for individual flies; horizontal line indicates the mean distance from center (mm). p values determined by Mann-Whitney *U*. The lower axis represents the effect size in Hedges’ *g* with 95% CI. Green circles and asterisk (^∗^) mark a statistically significant (p < 0.05) decrease in behavior. (D) Standardized mean effect sizes of diazepam effects on rodent anxiety (−0.85 *g* [95 CI −0.74, −0.96]) and fly WAFO (−0.83 *g* [95 CI −0.42, −0.91]) have comparable magnitudes. See also Figure S1.

Flies in enclosed chambers spent a large proportion of time near the walls (Figures 1 and S1) [18, 19]. While flies were able to crawl on all surfaces—floor, walls, and ceiling (Figure 1A)—cumulative locomotion traces were strikingly similar to rodent thigmotaxis data from open fields (Figure 1B) [14]. Flies on all surfaces were close to the wall, often 3–4 mm away from the center of a 5-mm chamber (Figure S1C). This behavioral feature, but not locomotion itself, was persistent (Figures S1D–S1F). We termed this behavior “wall following” (WAFO).

### Diazepam Reduces Fly Wall Following

Benzodiazepines reduce anxiety by modulating GABA_A_ receptors [20], and their binding site is evolutionarily conserved [21]. Diazepam reduces anxiety in three important rodent defense behavior assays: the open field (OF), the elevated plus maze (EPM), and the light/dark box [11]. In flies, diazepam had a pronounced effect on fly WAFO at three doses (Figure 1C). Raw behavioral metrics may have an indirect relationship to internal state and are not comparable across diverse experimental systems, for example, between different assays in distinct species. To contextualize the diazepam result, we calculated a standardized effect size (Hedges’ *g*) from the diazepam-induced WAFO change (Figure 1C, lower panel) and compared it with a meta-analytic rodent anxiety diazepam effect size calculated from 382 published rodent experiments (http://dx.doi.org/10.1101/020701). Diazepam effect sizes in both systems were comparable (Figure 1D).

### Altering *d5-HT1B* Function Has WAFO Effects that Are Concordant with Mouse Anxiety

**Figure 2:**
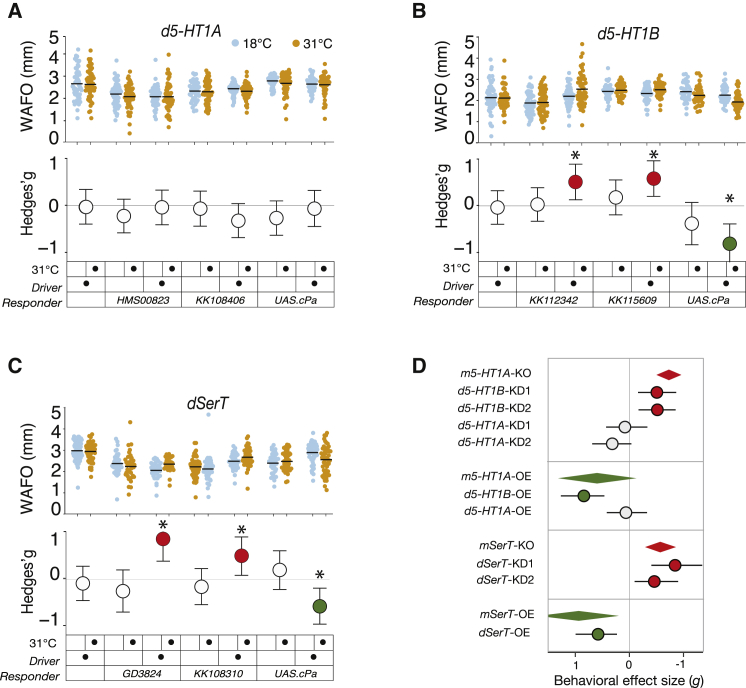
Anxiety-Concordant Effects of Serotonin Gene Lesions on Fly WAFO (A) Genetic lesions of *d5-HT1A* produced only minor effects in WAFO. Blue dots are untreated flies; orange dots are pre-warmed to 31°C as for GAL80^ts^ derepression. The lower axes show Hedges’ *g*; responder alleles are named in the boxes. The driver is *nSyb-Gal4, Tub-Gal80*^*ts*^. (B) Genetic lesions of *d5-HT1B* had moderate and statistically significant effects on WAFO: knockdown caused increases (*d5-HT1B*^*KK112342*^*g* = 0.51, p = 9 × 10^−3^; *d5-HT1B*^*KK115609*^*g* = 0.58, p = 2 × 10^−3^), while overexpression elicited a decrease (*g* = −0.82, p = 7.4 × 10^−5^, n = 53, 54). Red and green circles indicate a statistically significant WAFO change. (C) Knockdowns of *mSerT* with two RNAi lines produced consistent WAFO increases (*SerT*^*GD3824*^*g* = 0.63, p = 8.2 × 10^−4^, n = 60, 55; *SerT*^*KK108310*^*g2* = 0.48, p = 0.2 × 10^−2^, n = 60, 40), and overexpression decreased WAFO (*dSerT*^*Scer*∖*UAS*^·^*cPa*^*g* = −0.53, p = 1.8 × 10^−3^, n = 73, 75). Warm-treated controls for *d5-HT1A*, *d5-HT1B*, and *mSerT* UAS transgenes underwent modest, non-statistically significant changes. (D) A comparison of mouse anxiety gene effect sizes and fly ortholog WAFO effect sizes indicates they are concordant in direction and magnitude, except for *d5-HT1A* knockdowns. Diamonds indicate averaged meta-analytic values; circles indicate fly WAFO effect; lateral vertices and error lines are 95% CI. See also Figure S2.

Genetic experiments in mouse previously demonstrated that deleting and overexpressing the gene for the mammalian 5-HT1A receptor (m5-HT1A) produced moderate effects on rodent anxiety (http://dx.doi.org/10.1101/020701). *Drosophila* has two serotonin class 1 receptor genes with similarity to *m5-HT1A: d5-HT1A* and *d5-HT1B*. The function of these genes was knocked down in adult flies with lines expressing RNAi under the control of a warm-induced pan-neuronal driver, *nSyb-Gal4, tub-Gal80*^*ts*^ [22, 23]. Alterations of *d5-HT1A* expression with two RNAi lines and one cDNA responder produced only minor changes in WAFO (Figure 2A). However, the use of RNAi and overexpression to alter levels of *d5-HT1B* produced pronounced effects on WAFO (Figure 2B). These *d5-HT1B* effect sizes were of a comparable magnitude to the mouse anxiety effects from *m5-HT1A* lesions (Figure 2D) (http://dx.doi.org/10.1101/020701). Control experiments with warm treatment of control flies had trivial WAFO effects (Figures 2A and 2B). We conclude that manipulating *d5-HT1B* function influences fly WAFO in ways that parallel the effects that altering *m5-HT1A* expression has on mouse defense behaviors.

### Concordant *SERT* Effects on Fly WAFO and Mouse Anxiety

Deletion of *mSert* produces an increase in mouse anxiety (http://dx.doi.org/10.1101/020701). In flies, reducing *dSerT* mRNA levels with either of two RNAi alleles increased WAFO (Figure 2C). Flies expressing transgenic *dSerT* at 12× elevated levels (Figure S2P) had lowered WAFO (*g =* −0.53; Figure 2E), echoing the low anxiety observed in mice expressing elevated *mSert* (http://dx.doi.org/10.1101/020701). Control, warm-treated flies underwent no WAFO change (Figure 2C).

### Concordant Stress Effects on Fly WAFO and Mouse Anxiety

**Figure 3:**
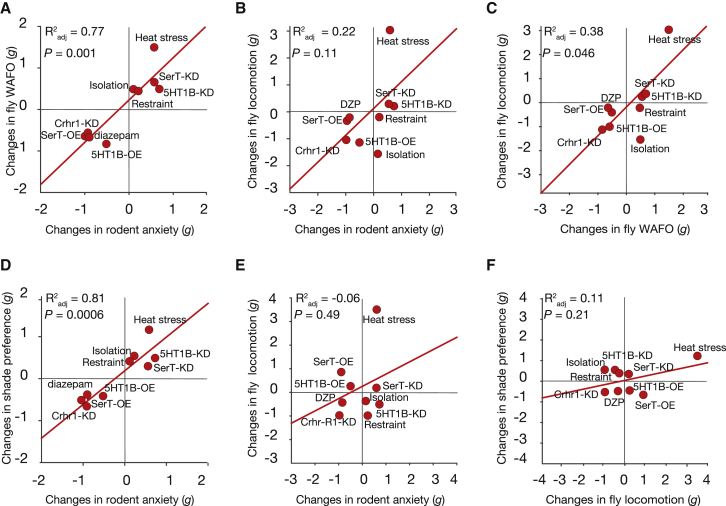
Fly Defense Behavior Outcomes Are Concordant with Anxiety Outcomes (A) A strong correlation between rodent anxiety and fly WAFO data for nine comparable manipulations (R^2^_adj_ = 0.77 [95 CI 0.58, 0.83]). The horizontal axis shows rodent meta-analytic *g* values; the vertical axis displays fly WAFO *g* values. The red line is the least-squares fit; p is for the F statistic of the model. (B) Walking speed changes in the square arena are weakly correlated with rodent anxiety outcomes (R^2^_adj_ = 0.22 [95 CI 0.0, 0.30]). (C) WAFO is moderately related to locomotion in the square arena (R^2^_adj_ = 0.38 [95 CI 0.06, 0.49]). (D) Light/dark choice outcomes are strongly correlated with rodent effect sizes (R^2^_adj_ = 0.81 [95 CI 0.64, 0.86]). (E) Changes in locomotion in the light/dark arena are weakly correlated with rodent anxiety outcomes (R^2^_adj_ = 0.06 [95 CI 0.0, 0.09]). (F) Light/dark choice outcomes are poorly correlated with locomotion (R^2^_adj_ = 0.11 [95 CI 0.0, 0.14]). See also Figure S3.

Environmental stress drives anxiety [24]. Subjecting flies to heat shock stress elicited a large WAFO increase (Figure 3A), concordant with the effect of acute pain on rodent anxiety (Figure S3D) ([25]; http://dx.doi.org/10.1101/020701). Diazepam reduced WAFO in heat-stressed flies, much as it did for flies at 25°C (Figures S2Q and S2R). Physically restraining flies produced a WAFO increase that was concordant with the anxiogenic effect of restraint in rodents (Figure S2E) (http://dx.doi.org/10.1101/020701). Ten days of social isolation stress increased fly WAFO (Figure S2F), an outcome that is concordant with isolation’s effect on rodent anxiety (http://dx.doi.org/10.1101/020701). The corticotropin-releasing hormone receptor 1 (CRHR1) is associated with mammalian stress, and knockout mice have lower anxiety; the fly homolog is the diuretic hormone 44 receptor 1 (DH44-R1) [26, 27]. Reducing *Dh44-R1* expression (Figure S2O) reduced WAFO (Figure S2K), consistent with mouse data (http://dx.doi.org/10.1101/020701). Interestingly, *Dh44-R1* mRNA levels were dramatically altered by all three stressors (Figure S4D).

### Anxiotropic Manipulations Influence *Drosophila* Light/Dark Choice

A second fly shelter/exposure assay with anxiety concordance would verify that exposure avoidance correlates with fly anxiety. The rodent light/dark choice assay examines light avoidance [12]. We used a simple chamber (Figure S3A) to measure changes in fly light/dark choice in response to anxiety manipulations. Of nine interventions, six (diazepam, *d5-HT1B* loss of function, *dSerT* overexpression, heat, restraint, and social isolation) had substantial statistical effects that were concordant with rodent anxiety data (Figure S3H). The other three were also directionally concordant but had modest, non-statistically significant effects on light/dark choice (*d5-HT1B* overexpression, *dSerT* knockdown, and *Dh44-R1* knockdown). These data largely verify the hypothesis that exposure avoidance measures a fly anxiety state.

### Effects in *Drosophila* WAFO and Light/Dark Choice Are Predictive of Rodent Anxiety Effects

Fly and rodent effect sizes for all interventions were subjected to cross-species linear regression. The regression models indicated that fly ΔWAFO data are largely predictive of rodent anxiety changes (R^2^_adj_ = 0.77 95% confidence interval [95 CI 0.47, 0.75]), as are the fly Δlight/dark choice outcomes (R^2^_adj_ = 0.81 [95 CI 0.58, 0.82]; Figures 3A and 3D). These results are compatible with the hypothesis that fly WAFO and fly light/dark choice, like rodent anxiety assays, test an anxiety-related brain state.

### Fly Defense Behaviors Are Distinct from Motor Activity

Motor activity and anxiety behavior are related phenotypes. Tranquilizers like diazepam also have sedative effects, and such overlap might also apply to neurogenetic systems. If WAFO and/or light/dark choice changes were purely a result of speed changes, this would erode confidence in their specificity to anxiety. However, this was not the case. Walking speed was altered in ways that were dissociated from WAFO (Figures S1F, S2A–S2C, and S2H–S2K). Individual flies’ WAFO metrics were poorly correlated with “raw” walking speed (WAFO-locomotion R^2^_adj_ = 0.18 [95 CI 0.17, 0.19], p = 1.0 × 10^−91^, n = 2,046), as were their light/dark preferences (shade preference-locomotion R^2^_adj_ = 0.05 [95 CI 0.04, 0.06], p = 1.0 × 10^−13^, n = 1,138). Additional regression analyses of fly walking speed changes (Δspeed) indicated that these could explain less than four-tenths of WAFO change variance (ΔWAFO; Figure 3C) and only a tenth of Δlight/dark variance (Figure 3F). Cross-species analyses indicated that fly speed changes were weakly predictive of rodent anxiety: only a fifth (WAFO; Figure 3B) and 6% (light/dark; Figure 3E) of variance was explained. Thus, while locomotor changes contribute to ΔWAFO and Δlight/dark choice, they are not the main driver.

### Identification of *5-HT2B, tsr, tmod, CCKLR-17D1*, and *CCKLR-17D3* as Fly Anxiety Factors

**Figure 4:**
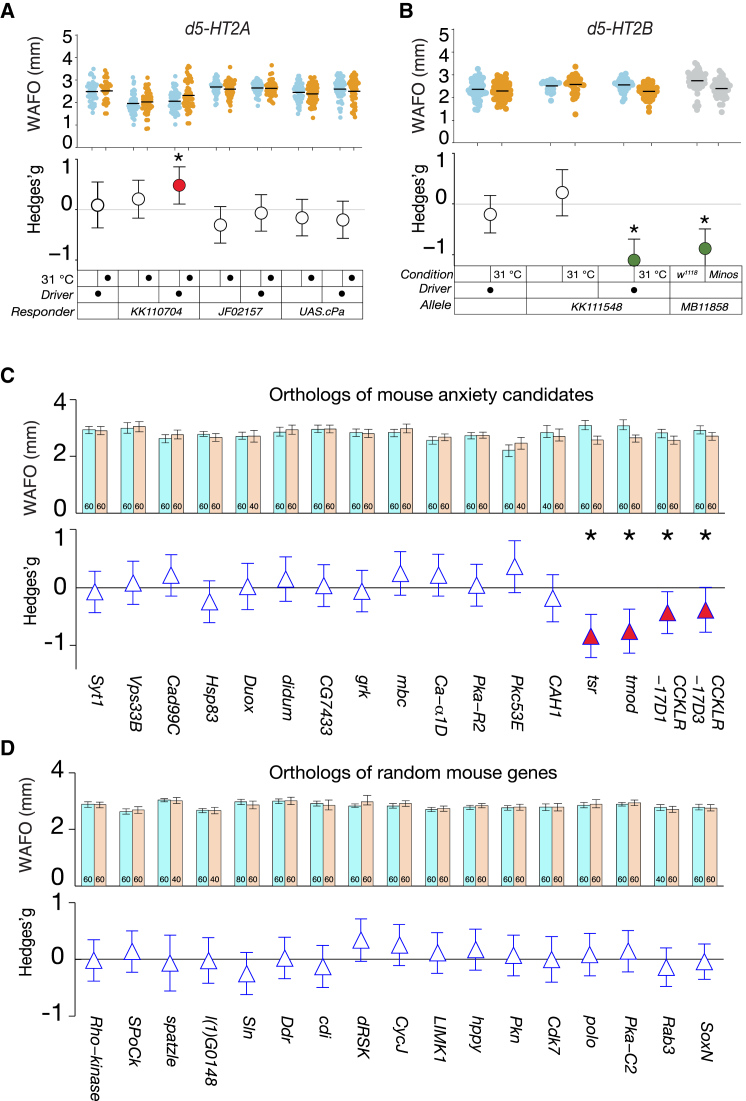
Identification of Candidate Fly Anxiety Genes (A) RNAi knockdown with *d5-HT2A*^*KK110704*^ increased WAFO (*g* = 0.48, p = 1 × 10^−2^), but this effect was not confirmed by a second RNAi allele (*d5-HT2A*^JF02157^*g* = −0.07, p = 6.9 × 10^−1^) or overexpression (*d5-HT2A*^*Scer∖UAS.cPa*^*g* = −0.21, p = 0.28). Warm-treated controls underwent non-statistical WAFO alterations. (B) Knockdown of *d5-HT2B* with *d5-HT2B*^*KK111548*^ produced a decrease in WAFO (*g* = −1.1, p = 6.8 × 10^−08^) as did a *Minos* transposon insertion into the gene: *d-HT2B*^*MB11858*^ (*g* = −0.88, p = 4.1 × 10^−06^). (C) Orthologs of candidate mouse anxiety genes were knocked down in the adult fly and tested for WAFO changes. Four knockdowns produced statistically significant reductions in WAFO: *tsr*^*KK108706*^ (*g* = −0.89, p = 5.0 × 10^−6^); *tmod*^*KK108701*^ (*g* = −0.81, p = 1.8 × 10^−5^); *CCKLR−17D1*^*KK108482*^ (*g* = −0.45, p = 3.5 × 10^−4^); and *CCKLR−17D3*^*KK110484*^ (*g* = −0.40, p = 1.2 × 10^−2^). Sample sizes are indicated at the base of the bars. (D) Seventeen randomly selected orthologs’ knockdowns had trivial effects on WAFO. See also Figure S4.

Wall following assays were used to identify fly anxiety gene candidates. Systematic review found that serotonin class 2 receptor knockouts have not been tested for their mouse anxiety role (http://dx.doi.org/10.1101/020701). Functional alterations of the two fly class 2 receptor genes, *d5-HT2A* and *d5-HT2B*, found that only the latter had consistent, substantial effects on WAFO (Figure 4B). Fly orthologs of candidate anxiety genes found at quantitative trait loci (QTLs) identified from a mouse genetic experiment were screened [28]. Of 17 genes, four showed WAFO alterations: *twinstar* (*tsr*), two *Cholecystokinin-like receptor* genes (*CCKLR-17D3* and *CCKLR-17D1*), and *tropomodulin* (*tmod*) (Figure 4), which are homologs of mouse *cofilin 1* (*Cfl1*), *cholecystokinin B receptor* (*Cckbr*), and *Tropomodulin-2* (*Tmod2*), respectively. Control tests of 17 randomly selected orthologs found none produced WAFO effects (Figure 4D). Interestingly, two mouse orthologs of the four fly anxiety candidate genes are known to anxiety research. *Cofilin-1* is a mouse anxiety gene with a knockout having a concordant outcome to the fly WAFO result [28]. Mouse *Cckbr* codes for cholecystokinin receptor, and its deletion has an effect concordant with knockdown effects of fly WAFO [29].

## Discussion

The results verify the hypothesis that exposure avoidance behaviors of *Drosophila* share underlying neurogenetic pathways with mammalian anxiety. A GABA-modulating drug, serotonin receptor and transporter alterations, a stress peptide receptor, and environmental stressors produced effects that were concordant with comparable manipulations in mammalian anxiety-related behaviors. A regression comparison of fly behavior data and rodent anxiety data indicated that the two are similar. The high coefficients of determination observed in the interspecies comparisons are remarkable in that they would not be expected to account for sources of variance that include sampling error, within- and between-lab heterogeneity, publication bias, >600 million years of evolutionary divergence, or the difference between semi-acute knockdowns and lifelong knockouts.

A candidate survey newly implicated *d5-HT2B*, *tsr*, *tmod*, *CCKLR-17D3*, and *CCKLR-17D1* in fly anxiety. The anxiolytic effect of *tsr* supports the hypothesis that actin microfilament stability is connected to anxiety [28], consistent with ideas that actin polymerization influences anxiety via aversive memory formation and stability [30] and/or related processes [31]. Similarly, that CCK-like receptor knockdowns reduce fly anxiety supports the hypothesis that CCK receptors are involved in anxiety and fear [32, 33], with a role proposed specifically for the mammalian cholecystokinin B receptor (CCKBR) [29]. In flies, the putative ligand for the CCKLR receptors is DROSULFAKININ (DSK); intriguingly, *CCKLR-17D1* and *dsk* mutants have deficits in a larval stress-induced escape behavior [34]. The implication of CCK-like receptors in fly defense behaviors suggests that this is an anxiety-related signaling system, like GABA, serotonin, and Dh44-R1/CRHR1. Most of the orthologous gene knockdowns produced no WAFO effect, suggesting that the QTL hits include false positives and that WAFO genes are relatively rare.

Anxiety research has struggled to find new therapeutics [11]. Bringing the neurogenetic tools and larger sample sizes of *Drosophila* to bear on anxiety promises to complement rodent model analysis of anxiety and anxiety disorders.

## Author Contributions

Conceptualization, F.M. and A.C.-C.; Methodology, F.M. and A.C.-C.; Software, F.M., S.A., J.H., J.C.S., and A.C.-C.; Investigation, F.M., T.L.T., N.A.N., and A.E.; Writing, F.M. and A.C.-C.; Visualization, F.M.; Supervision, A.C.-C.; Project Administration, A.C.-C.; Funding Acquisition, A.C.-C.

## Supplemental Information

Document S1. Supplemental Experimental Procedures and Figures S1–S4

Document S2. Article plus Supplemental Information
