# Supplementary material for: Ancient Anxiety Pathways Influence Drosophila Defense Behaviors
Source: Curr Biol. 2016 Apr 4;26(7):981–6. doi: 10.1016/j.cub.2016.02.031 (PMC4826436; doi:10.1016/j.cub.2016.02.031)
Supplement: Document S1. Supplemental Experimental Procedures and Figures S1–S4 [file mmc1.pdf]

Current Biology, Volume *26*

Supplemental Information

Ancient Anxiety Pathways Influence *Drosophila* Defense Behaviors

Farhan Mohammad, Sameer Aryal, Joses Ho, James Charles Stewart, Nurul Ayuni Norman, Teng Li Tan, Agnese Eisaka, and Adam Claridge-Chang

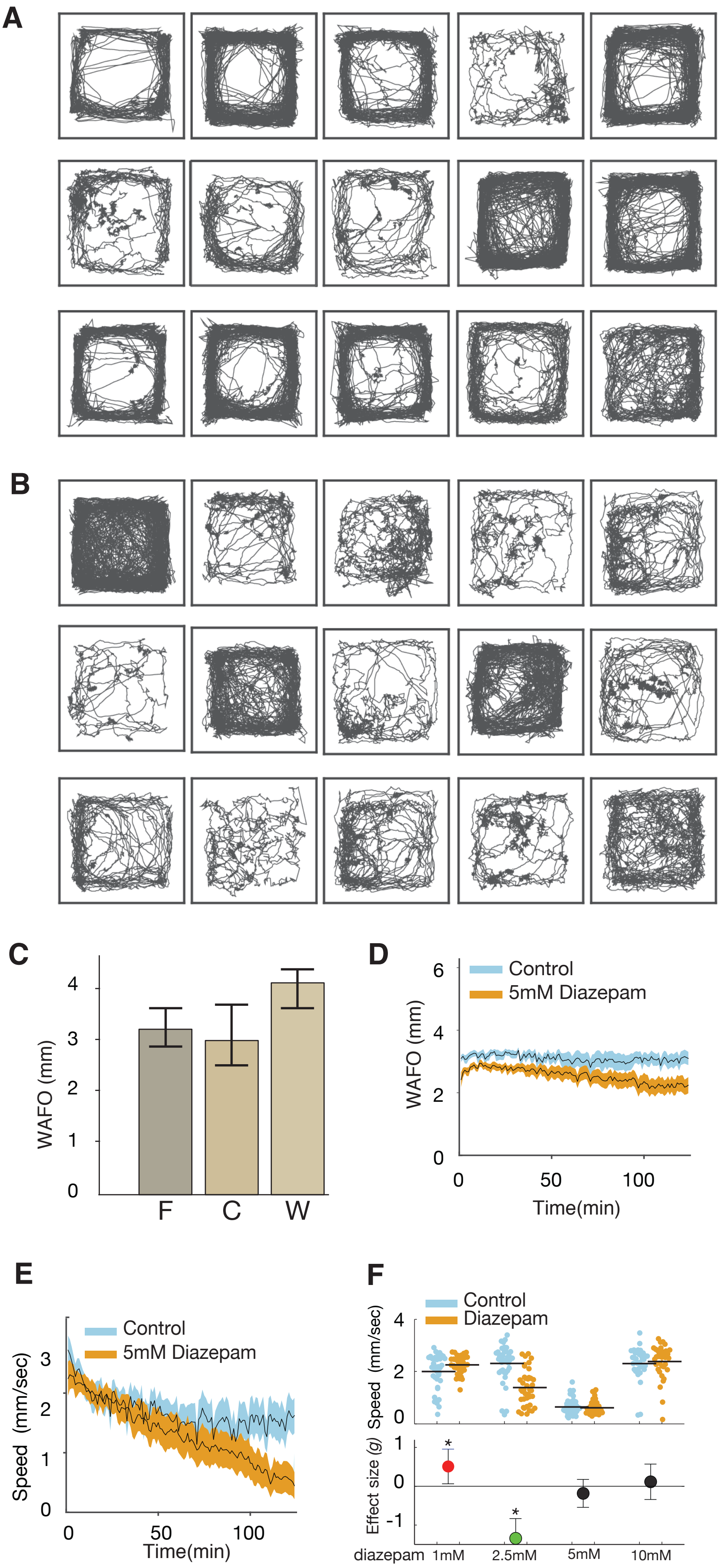
A
B
C
WAFO (mm)
0
1
2
3
4
F
C
W
D
Control
5mM Diazepam
WAFO (mm)
0
2
4
6
0
50
100
Time(min)
E
Control
5mM Diazepam
Speed (mm/sec)
0
1
2
3
0
50
100
Time(min)
F
Control
Diazepam
Speed (mm/sec)
Effect size (g)
4
2
0
1
0
-1
*
*
diazepam
1mM
2.5mM
5mM
10mM

Figure S1

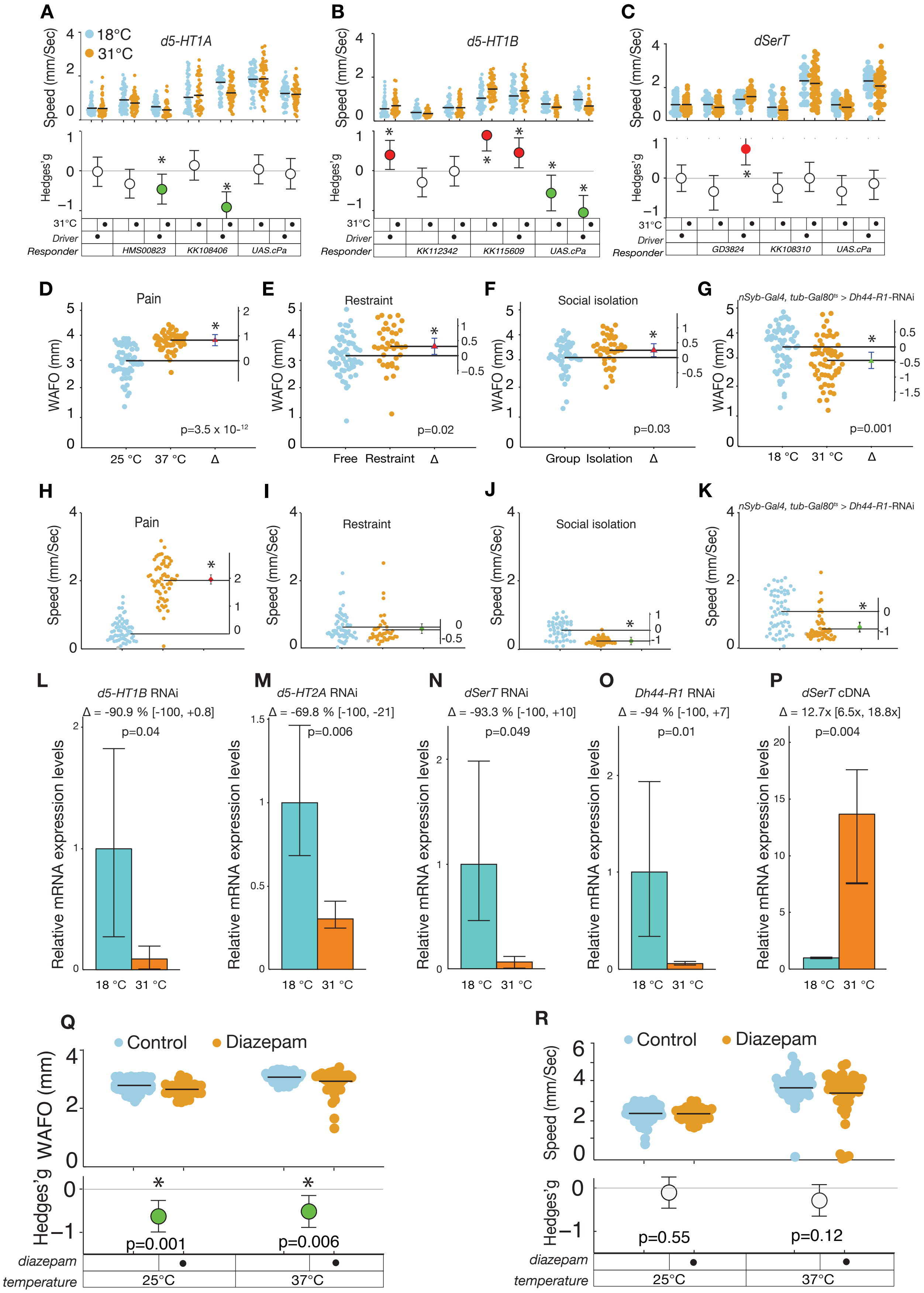

A
B
C
D
E
F
G
H
I
J
K
L
M
N
O
P
Q
R
d5-HT1A
d5-HT1B
dSerT
18°C
31°C
Speed (mm/Sec)
Hedges'g
31°C
Driver
Responder
HMS00823
KK108406
UAS.cPa
KK112342
KK115609
GD3824
KK108310
Pain
Restraint
Social isolation
nSyb-Gal4, tub-Gal80ts > Dh44-R1-RNAi
WAFO (mm)
p=3.5 x 10-12
p=0.02
p=0.03
p=0.001
25 °C
37 °C
Free
Group
Isolation
18 °C
31 °C
d5-HT1B RNAi
Δ = -90.9 % [-100, +0.8]
p=0.04
d5-HT2A RNAi
Δ = -69.8 % [-100, -21]
p=0.006
dSerT RNAi
Δ = -93.3 % [-100, +10]
p=0.049
Dh44-R1 RNAi
Δ = -94 % [-100, +7]
p=0.01
dSerT cDNA
Δ = 12.7x [6.5x, 18.8x]
p=0.004
Relative mRNA expression levels
Control
Diazepam
p=0.001
p=0.006
p=0.55
p=0.12
diazepam
temperature
25°C
37°C

Figure S2.

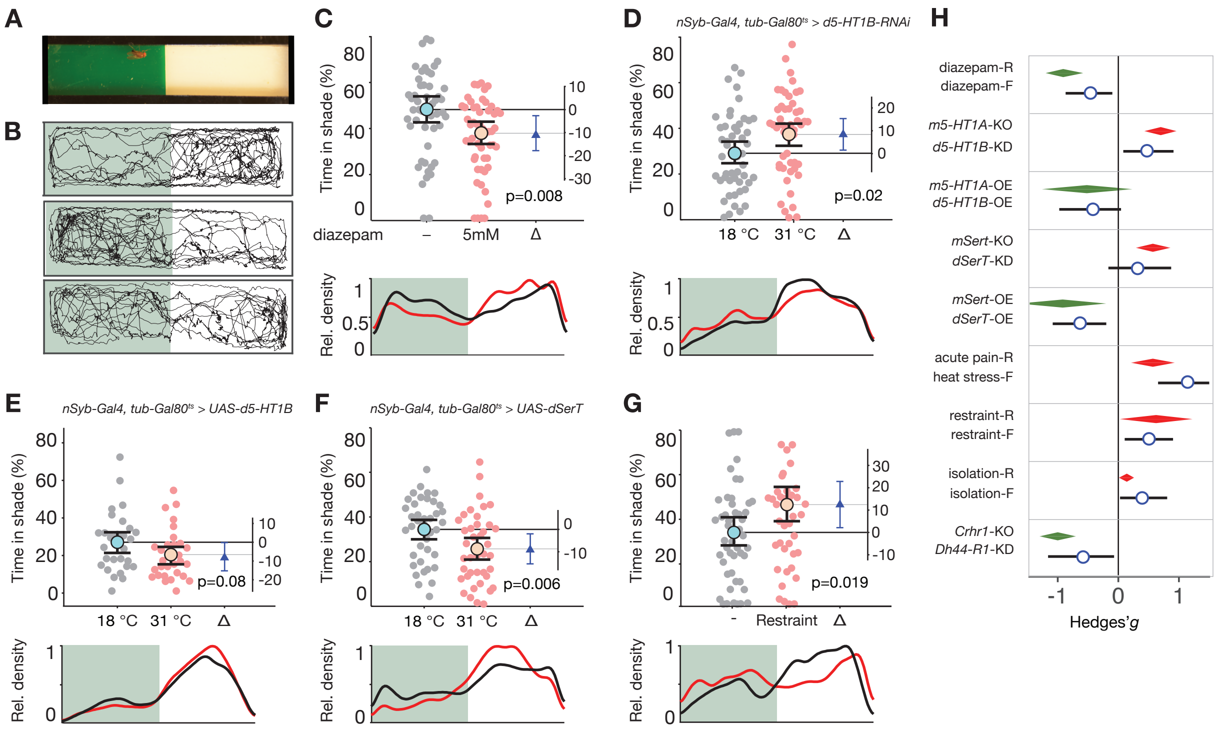

A
B
C
Time in shade (%)
80
60
40
20
0
10
0
-10
-20
-30
p=0.008
diazepam
–
5mM
Δ
Rel. density
1
0.5
0
D
nSyb-Gal4, tub-Gal80ts > d5-HT1B-RNAi
Time in shade (%)
20
10
0
p=0.02
18 °C
31 °C
Δ
E
nSyb-Gal4, tub-Gal80ts > UAS-d5-HT1B
p=0.08
F
nSyb-Gal4, tub-Gal80ts > UAS-dSerT
p=0.006
G
30
p=0.019
-
Restraint
H
diazepam-R
diazepam-F
m5-HT1A-KO
d5-HT1B-KD
m5-HT1A-OE
d5-HT1B-OE
mSert-KO
dSerT-KD
mSert-OE
dSerT-OE
acute pain-R
heat stress-F
restraint-R
restraint-F
isolation-R
isolation-F
Crhr1-KO
Dh44-R1-KD
-1
0
1
Hedges'g

Figure S3.

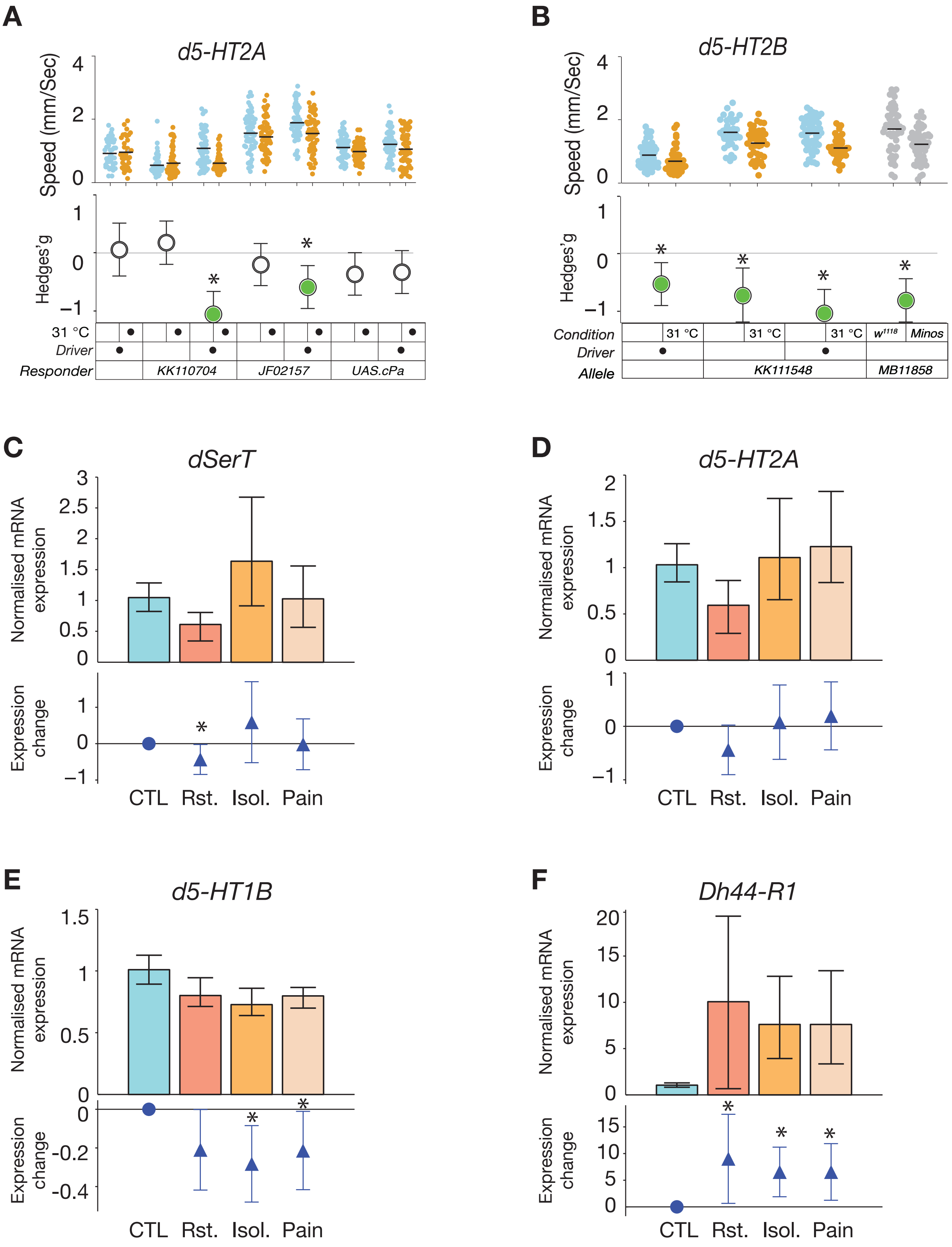
A
d5-HT2A
Speed (mm/Sec)
Hedges'g
31 °C
Driver
Responder
KK110704
JF02157
UAS.cPa
B
d5-HT2B
Speed (mm/Sec)
Hedges'g
Condition
31 °C
Driver
Allele
KK111548
MB11858
Minos
C
dSerT
Normalised mRNA expression
Expression change
CTL Rst. Isol. Pain
D
d5-HT2A
Normalised mRNA expression
Expression change
CTL Rst. Isol. Pain
E
d5-HT1B
Normalised mRNA expression
Expression change
CTL Rst. Isol. Pain
F
Dh44-R1
Normalised mRNA expression
Expression change
CTL Rst. Isol. Pain

Figure S4.

SUPPLEMENTAL INFORMATION

Supplemental Figure Legends

Figure S1. Locomotion data for flies treated with diazepam

Refers to Figure 1.

A. Example cumulative traces of the movement of 15 flies treated with solvent.

B. Traces of locomotor behavior of flies treated with diazepam (2.5 mM)

C. When their feet are contacting the floor (F), the ceiling (C), or walls (W) flies stay 4.1 mm [95CI 4.5, 3.6], 3.2 mm [95CI 3.6, 2.8], and 3.0 mm [95CI 3.6, 2.3] from the center, respectively.

D. Both diazepam treated and non-treated flies' WAFO (mean distance from the center) did not change appreciably over 120 min (N = 40 flies). Color shading is 95% confidence intervals.

E. Both diazepam treated and non-treated flies' walking speed slowed over time (N=40 flies). Statistical differences were observed after ~90 minutes.

F. Some diazepam-fed flies have large differences in walking speed; in these experiments, locomotion was statistically increased or decreased in flies fed 1 mM or 2.5 mM, respectively, but this was not conventionally dose-dependent; higher concentrations did not elicit statistical changes.

Figure S2. The effects of receptor gene lesions and stress on WAFO and locomotion

Refers to Figure 2.

A. Knockdown of the *d5-HT1A* receptor with RNAi reduces locomotion, but overexpression has a trivial effect.

B. Locomotion is statistically altered in five experiments with the *d5-HT1B* receptor allele, but these are not attributable to gene knockdown effects as changes are seen in driver-transgene-only control flies subjected to warm treatment.

C. Locomotion is statistically altered in only one *dSerT* experiment.

D. Exposing flies to 37°C dramatically increased proximity to the wall; $g = 1.4$, $P = 3.5 \times 10^{-12}$. The P values in this figure were calculated using the Mann–Whitney *U* method.

E. One hour of restraint stress in a nested pipette tip increased WAFO in flies tested after this treatment, compared with control animals allowed to move freely: $g = 0.41$, $P = 2.4 \times 10^{-2}$, N = 58, 39.

F. Isolating Canton-S male flies from social contact for 10 days led to increased WAFO: $g = 0.475$, P = 0.029, N = 60, 40.

G. Knockdown of *Dh44-R1* with $Dh44\text{-}R1^{KK108591}$ resulted in decreased WAFO: $g = -0.57$, $P = 1.0 \times 10^{-3}$, N = 60, 59.

H. Stressful heat at 37°C has a large effect on walking speed in the square arena (g = 3.1, P = 2.7 × 10^{-19}, N = 60 60).

I. Restraint stress has a negligible effect on walking speed (g = -0.21, P = 4 × 10^{-2}, N = 58, 39).

J. Social isolation suppressed walking speed (g = -1.6, P = 9 × 10^{-9}, N = 49, 40).

K. Knockdown of Dh44-R1 lowers walking speed (g = -1.03, P = 1.2 × 10^{-6}, N = 60, 59).

L. Quantitative PCR of transgenic *Drosophila* lines bearing RNAi or cDNA alleles verifies the effects on mRNA expression. Flies were assayed for mRNA levels after treatment with a protocol identical to the one used for induction prior to behavior. Targeting the *d5−HT1B* receptor gene with $d5{-}HT1B^{KK112342}$ led to a 91% reduction of the fly head mRNA level (g = −1.2, P = 0.047, N = 5, 5 experiments). The blue bars are normalized expression levels in uninduced flies, the orange bars are data from induced flies. The confidence interval of each percent change was calculated by bootstrap and P was calculated with the Mann-Whitney-Wilcoxon *U* method.

M. Targeting *d5−HT2A* with $d5{-}HT2A^{KK110704}$ led to a 70% reduction of expression (g = −1.7, P = 0.006, N = 6, 6).

N. Targeting *SerT* with $SerT^{GD3824}$ for three successive nights (~17 hours each) at 31 °C led to a 93% reduction in mRNA (g = −1.2, P = 0.050, N = 3, 3).

O. Induction of RNAi from the $Dh44{-}R1^{KK108591}$ allele led to a 94% reduction in mRNA (g = −0.84, P = 0.027, N = 4, 5).

P. Induction of the $SerT^{Scer-UAS.cPa}$ transgene led to a 12.7-fold increase in mRNA levels (g = 2.4, P = 0.004, N = 6, 6).

Q. Flies fed with 5 mM diazepam show reduced WAFO at both 25°C (g = −0.63, p = 0.001, N = 60, 60) and stressful 37°C (g = −0.52, p = 0.006, N = 60, 60).

R. Diazepam 5 mM has a minimal effect on locomotion at 25°C (g = −0.11, p = 0.55, N = 60,60) and modest reduction at 37°C (g = −0.29, p = 0.12, N = 60,60).

Figure S3: A light/dark choice assay for *Drosophila* and anxiety manipulations

Refers to Figure 3.

A. A photograph of a light/dark choice chamber in which one half is shaded by a green filter.

B. Representative cumulative traces of flies moving in light/dark chambers for 10 minutes.

C. Starved wild type flies fed 5 mM diazepam displayed decreased time spent on the shaded side (g = -0.52, P = 0.008, N = 52, 55) relative to flies fed the carrier liquid (5% sucrose, 20% v/v Tween-80) alone. The lower panel shows the relative density of fly locations for all video frames from all 107 flies without (black) and with diazepam treatment (red), dual normalized histograms in which the maximal density value is set to 1. Green box indicates the shaded area. All P values were calculated by the Mann–Whitney *U* method.

D. Flies targeted with an RNAi against *d5-HT1B* spent an increased amount of time in the dark shaded area: $g = 0.47$, P = 0.02, N = 50, 50.

E. Flies carrying an induced *d5-HT1B* cDNA transgene showed decreased time spent in the shaded region: $g = -0.449$, P = 0.08, N = 30, 30, not statistically significant

F. Flies with overexpressed *dSerT* decreased the time they spent in the shaded region: $g = -0.62$, P = 0.0067, N = 40, 40.

G. Exposing Canton-S flies to one hour of restraint stress increased time spent in the shaded region: $g = 0.49$, P = 0.019, N = 50, 46.

H. Comparison of effect sizes from meta-analyses of published rodent data (diamonds) with corresponding fly light/dark assay results (blue). The fly primary data are compatible with the rodent meta-analytic data, except for isolation (which has the same direction, but is substantially smaller in fly).

Figure S4: The effects of serotonin class 2 receptor gene lesions on locomotion and stress-related anxiety gene expression.

Refers to Figure 4.

A. The *d5-HT2A* knockdowns reduced walking speed, but overexpression had little effect.

B. Reducing function of *d5-HT2B* lowered walking speed in all experiments, but this could not be attributed to the gene function as controls were also affected.

C. Serotonin transporter (*dSerT*) mRNA levels were statistically unchanged by diverse stressors: restraint Rst., P = 0.11, N = 6, 6; isolation Isol., P = 0.34, N = 6, 6; heat shock at 37°C (P = 0.10, N = 6, 5). Flies were subjected to one of three stressors prior to being assayed by qPCR for expression levels relative to untreated flies (CTL). For each experiment, the top panel indicates expression relative to control flies and the bottom panel plots the expression fold change and its 95% confidence intervals. Confidence interval bars of the contrast (change) indicate statistical significance when they do not cross the zero line (this is also indicated by the red asterisks). All P values in this figure were calculated using the Mann–Whitney–Wilcoxon *U* method.

D. *d5-HT2A* mRNA remained statistically unchanged in response to any of the stressors: restraint (FC = -0.44, p = 0.88,, N = 6, 4), isolation (= 0.0.08, p = 0.1, N = 6, 6), heat (*FC* = 0.19, p = 0.71, N = 6, 5).

E. *d5-HT1B* mRNA in fly heads is downregulated in response to all three stressors. Exposing flies to one hour of restraint stress reduced *d5-HT1B* mRNA levels (−0.21 × decrease, P = 0.14, N = 6, 5), although this was not statistically significant. Housing male flies in isolation lowered *d5-HT1B* levels (−0.28 × decrease, P = 0.016, N = 6, 6). Exposing flies to 37°C for 10 minutes also decreased *d5-HT1B* mRNA (−0.21 × decrease, P = 0.01, N = 6, 4).

F. *Dh44-R1* mRNA was dramatically elevated by all three stressors: restraint (9 × increase [95CI 3, 12]; N = 6, 4; P = 0.01), isolation (6.6 × increase [95CI 3, 11]; N = 6, 4; P = 0.01) and stressful heat (6.5 × increase [95CI 2, 19]; N = 6, 3; P = 0.03).

Supplemental experimental procedures

Fly strains

Flies were cultured and maintained on fly medium at 24°C and 60% humidity on a 12 h light: 12 h dark cycle unless otherwise mentioned. Wild-type stocks were *w*^{1118} and Canton-Special (C-S). Strains carrying inverted repeat RNAi transgenes were obtained from the Vienna Drosophila Research Centre (VDRC) and Bloomington Drosophila Stock Center (BDSC). The *UAS-RNAi* lines used were: *d5-HT1A*^{KK108407}, *d5-HT1A*^{HMS00823}, *d5-HT1B*$^{\mathrm{KK112342}}$(v109929), *d5-HT1B*$^{\mathrm{KK115609}}$(v110128), *d5-HT2A*$^{\mathrm{KK110704}}$ (v102105), *d5-HT2A*(JF02157), *d5-HT2B*^{KK111548} (v102356), *d-HT2B*^{MB11858}, *SerT*$^{\mathrm{KK108310}}$ (v100584), *SerT*$^{\mathrm{GD3824}}$ (v11346), and *Dh44-R1*$^{\mathrm{KK108591}}$ (v110708). UAS lines were obtained from the Bloomington Drosophila Stock Center (BDSC): *SerT*$^{Scer\backslash UAS.cPa}$ [S1], *d5-HT1A*$^{Scer\backslash UAS.cPa}$ and *d5-HT1B*$^{Scer\backslash UAS.cPa}$ [S2], and 5-HT2$^{Scer\backslash UAS.cPa}$ [S3]. *nSyb-Gal4, Tub-Gal80*^{ts} conditional pan-neural driver strain was generated by standard methods from the constituent transgenics: *nSyb-GAL4* was a gift from Bassem Hassan and *Tub-Gal80*^{ts} was acquired from BDSC.

Transgenic animal preparation

For temperature-controlled transgene expression [S4], *nSyb-Gal4, tub-Gal80*^{ts}*/TM3* virgin females were crossed with males carrying a responder transgene (*i.e. UAS-RNAi* or *UAS-cDNA)* [S5]. This combination allowed temporal control of neural expression using thermal de-repression of GAL80^{ts} inhibition of GAL4-activated transcription at 31°C. We used comparisons between isogenic fly groups so as to avoid possible effects from strain genetic background and temporal control to exclude developmental effects. The progeny of these crosses were raised at 18°C to maintain repression of the responder gene. Newly eclosed non-*TM3* males were isolated and maintained as a group of 24 flies in vials (2.4 cm diameter × 9.4 cm height) for 2–3 days before the behavioral assays. Vials of these animals were either maintained at 18°C until assaying, or treated to one or more overnight (~16 h) interval/s at 31°C. A single night of induction was initially used for each responder. In the case of *dSerT*^{GD3824}, no behavioral effect was seen with 1 night induction so the experiment was repeated with 3 consecutive nights at 31°C, with days at 18°C. Prior to the behavioral assay, flies were held for a 2–3 h recovery period at 25°C. In addition to the non-induced flies carrying *UAS* transgenes, further control animals were generated by crossing the driver line with wild type flies (either C-S or a *w*^{1118} strain received from VDRC) and raising the progeny under identical regimes as the flies carrying the responder transgenes. *d-HT2B*^{MB11858} line was first outcrossed to W1118 background for 6 consecutive generations; control and experimental flies were raised together at identical conditions before assaying them for WAFO at 25°C.

Candidate anxiety gene screen

The following VDRC RNAi alleles were used to assess the orthologs of the candidate anxiety genes from the mouse QTL study: *Syt1*^{KK108653}, *Vps33B*^{GD14789} , *Cad99C*^{GD153}, *Hsp83*^{KK101256}, *Duox*^{GD844}, *didum*^{GD1848}, *CG7433*^{GD12238}, *grk*^{KK105496}, *mbc*^{GD6965}, *Ca-alpha1D*^{GD1737}, *Pka-R2*^{KK109446}, *Pkc53E*^{GD11984}, *CAH1*^{KK108727}. For the randomly selected genes, the following VDRC RNAi alleles were used: *Rho-Kinase*^{KK107802}, *Spock*^{GD15076}, *Spatzle*^{KK112908}, *l(1)G0148*^{GD13811}, *SoxN*^{GD4415}, *Rab3*^{KK108633}, *Pka−C2*^{GD4649}, *polo*^{GD7563}, *Cdk7*^{GD4167}, *Pkn*^{KK101337}, *hppy*^{GD12129}, *LIMK1*^{GD9586}, *CycJ*^{GD6936}, *dRSK*^{KK109199}, *cdi*^{GD8731}, *Ddr*^{GD13382}, *Sln*^{KK104306}.

Diazepam treatment

Diazepam was dissolved in 100% ethanol to prepare a stock solution. Diazepam stock solution was diluted in 5% sucrose and 5% yeast-extract solution to prepare solutions corresponding to dosages of 2.5–10 mM. Overnight starved flies were fed with diazepam solutions for ~6-8 h using capillary feeders (CAFE) [S6] at 25°C prior to behavioral testing. Red dye in the food was used to monitor drugged food consumption in the flies. Non-feeding flies were excluded from the analysis. Control flies were fed with carrier solutions mixed with an equivalent amount of ethanol. An identical quantity of ethanol was used across diazepam concentrations, i.e. ethanol was held constant.

Stressors

C-S male flies were used in all stress paradigms. Heat shock was induced by heating the flies to 37°C while in the arena. For restraint stress, each individual fly was immobilized in a small space formed between two nested 10 µl pipette tips (Eppendorf). This arrangement allowed ventilation through the tips but prevented most movement. Flies were restrained for a period of one hour prior to behavioral testing. For single housing, C-S pupae were removed from vials and placed into vials either singly or in groups. Once eclosed, individual males flies were retrieved and transferred to either a second isolation vial or a vial with 24 other males for ten days prior to the assay.

Behavioral assays

For each behavioral assay, batches of 20–25 male flies were isolated under carbon dioxide anesthesia and placed in food vials 2–3 days prior to behavioral recording. Just prior to assay, a batch was knocked into an ice-chilled vial for 20–30 seconds (cold anesthesia) before they were placed individually into an arena with forceps. The wall-following arena was a 1 cm square with a height of 1.6 mm; twenty arenas were laser cut from a sheet of black acrylic to allow simultaneous video analysis of multiple flies. The light/dark box was a 40 × 4 mm rectangular arena; ten such arenas were cut from an acrylic sheet. Each arena was equally divided into two 20 × 4 mm illuminated and dimmed light arenas using transparent plastic sheet on one side and transparent green filter on the

other side. The individual array was covered with a transparent, anti-reflection coated glass sheet (Edmund optics) and was placed in an incubator. The open field was lit from the sides and the light/dark box from below by white LED microscope lamps (Falcon Illumination). To image the flies, an AVT Guppy F-046B CCD camera (Stemmer Imaging) equipped with a 12 mm CCTV-type lens was positioned above the behavioral arenas and connected to a computer via a IEEE 1394 cable. Flies were allowed to freely explore the arena during a 10-minute test session. Recordings were taken for 10 minutes in all assays following similar protocols in rodent and fly [S7-10]. Flies' positions were determined from a live video feed with CRITTA, custom software written in LabVIEW (National Instruments), which extracted centroid x-y position data and recording it to a binary file for offline analysis in MATLAB. For almost all experiments, samples sizes were approximately N = 60, 60, so as to yield a statistical power of ~0.75 when alpha = 0.05, assuming a moderate effect size of 0.5 standard deviations. All sample sizes were precisely N = 60, 60, unless otherwise mentioned. Behavioral tests of control and intervention flies were run in alternation on the same day.

Quantitative PCR

To measure mRNA levels in transgenic animals, batches of 2–3-day-old male flies were isolated from the crosses of *nSyb-Gal4*; *tub-Gal80ts/TM3* with the responder transgene (*i.e., UAS-RNAi* or *UAS-cDNA)* and were either maintained at 18°C or treated for 16–18 h overnight interval(s) at 31°C. For each gene expression analysis in response to stress, stressors were applied to 2–3-day-old C-S male flies. Heads of flies were separated using copper sieves, liquid nitrogen, and dry ice. Five independent total RNA samples were isolated immediately from different groups of control and stressors from ~20 heads for each sample using TriZol (Sigma), and stored at -80°C till further use. The RNA concentration was assessed using NanoDrop. From each condition, 2 µg of RNA was reverse transcribed into cDNA using a cDNA synthesis kit (Applied Biosystems). The expression of each gene was assayed in 96-well optical plates in a 7700 Sequence Detection System (Applied Biosystems) with default settings. All qPCR reactions were performed in duplicate and threshold cycle values (C_t) were averaged. TaqMan-based gene-specific primers were used to efficiently probe the respective gene transcripts. Normalized relative expression levels were calculated with *Rpl32* levels as the internal control. The relative quantification in gene expression was determined using the $2^{-\Delta\Delta Ct}$ method [S11].

Data analysis and statistics

Custom MATLAB scripts were used to analyze behavior and expression data. Fly centroid x-y location data were filtered to exclude unmoving, dead, or missing flies. The filtered dataset was used to calculate the distance from the arena's center in the WAFO assay and percent time spent in the shade in the light/dark choice assay. In the WAFO assay, we used mean distance from the center as

our raw measure of WAFO; this metric was preferred over the 'proportion of time spent in central zone' metric widely used in rodent studies as the latter relies on an arbitrarily defined central area and in our hands had a highly non-normal distribution. Contrasts were reported as standardized mean differences (Hedges' *g*). Hedges' *g* and its bootstrapped 95% confidence interval for the difference between control and experimental animals were calculated with the Measurements of Effect Size toolbox (MES) in MATLAB [S12]. Hedges' *g* is a standardized effect size expressed in units of standard deviation [S13]; specifically, *g* is the preferred variant of Cohen's d that uses SD_{pooled} and adjusts for bias [S14]. Standardized effect sizes have the benefit of allowing combinations across and comparisons between different experimental systems. Because behavioral assay metrics may have a non-linear and/or indirect relationship to internal brain state, standardizing the contrast effect size with the standard deviation has the additional benefit of recasting the data in terms of the controls' behavior, a superior reference point than the chamber size. Standardized effect sizes including Hedges' *g* are conventionally classified as 'trivial' (< 0.2 standard deviation), 'small' (< 0.5), 'medium' (< 0.8) and 'large' (> 0.8) {Borenstein:2011um). The total distance traveled and experimental duration (10 min) were used to calculate the average speed of the fly. Means and their 95% confidence intervals are reported in the format: 'mean [95CI lower bound, upper bound]'. Linear regression was done with the LinearModel.fit function in Matlab. For comparisons of two independent groups, confidence intervals of the primary data and the contrasts were calculated by the bias-corrected and accelerated (BCa) bootstrap method, and P was computed with the Mann-Whitney-Wilcoxon *U* method (using the bootci and ranksum functions respectively). All error bars in all data figures are 95% confidence intervals.

SUPPLEMENTAL REFERENCES

S1. Park, S. K., George, R., Cai, Y., Chang, H. Y., Krantz, D. E., Friggi-Grelin, F., Birman, S., and Hirsh, J. (2006). Cell-type-specific limitation on in vivo serotonin storage following ectopic expression of the Drosophila serotonin transporter, dSERT. J Neurobiol *66*, 452–462.

S2. Yuan, Q., Lin, F., Zheng, X., and Sehgal, A. (2005). Serotonin modulates circadian entrainment in *Drosophila*. Neuron *47*, 115–127.

S3. Colas, J. F., Launay, J. M., Vonesch, J. L., Hickel, P., and Maroteaux, L. (1999). Serotonin synchronises convergent extension of ectoderm with morphogenetic gastrulation movements in Drosophila. Mech Dev *87*, 77–91.

S4. McGuire, S. E., Le, P. T., Osborn, A. J., Matsumoto, K., and Davis, R. L. (2003). Spatiotemporal rescue of memory dysfunction in *Drosophila*. Science *302*, 1765–1768.

S5. Dietzl, G., Chen, D., Schnorrer, F., Su, K.-C., Barinova, Y., Fellner, M., Gasser, B., Kinsey, K., Oppel, S., Scheiblauer, S., et al. (2007). A genome-wide transgenic RNAi library for conditional gene inactivation in Drosophila. Nature *448*, 151–156.

S6. Ja, W. W., Carvalho, G. B., Mak, E. M., la Rosa, de, N. N., Fang, A. Y., Liong, J. C., Brummel, T., and Benzer, S. (2007). Prandiology of Drosophila and the CAFE assay. Proc Natl Acad Sci USA *104*, 8253–8256.

S7. Parks, C. L., Robinson, P. S., Sibille, E., Shenk, T., and Toth, M. (1998). Increased anxiety of mice lacking the serotonin1A receptor. Proc Natl Acad Sci USA *95*, 10734–10739.

S8. Liu, L., Davis, R. L., and Roman, G. (2007). Exploratory activity in *Drosophila* requires the kurtz nonvisual arrestin. Genetics *175*, 1197–1212.

S9. Gross, C., Zhuang, X., Stark, K., Ramboz, S., Oosting, R., Kirby, L., Santarelli, L., Beck, S., and Hen, R. (2002). Serotonin1A receptor acts during development to establish normal anxiety-like behaviour in the adult. Nature *416*, 396–400.

S10. Martin, J., Ernst, R., and Heisenberg, M. (1999). Temporal pattern of locomotor activity in Drosophila melanogaster. Journal of Comparative Physiology A: Sensory.

S11. Livak, K. J., and Schmittgen, T. D. (2001). Analysis of relative gene expression data using real-time quantitative PCR and the 2(-Delta Delta C(T)) Method. Methods *25*, 402–408.

S12. Hentschke, H., and Stüttgen, M. C. (2011). Computation of measures of effect size for neuroscience data sets. Eur J Neurosci *34*, 1887–1894.

S13. Cumming, G. (2012). Understanding the New Statistics: Effect Sizes, Confidence Intervals, and Meta-Analysis.

S14. Borenstein, M., Hedges, L. V., Higgins, J., and Rothstein, H. R. (2011). Introduction to meta-analysis (John Wiley & Sons, Ltd).
