# Supplementary material for: Ancient Anxiety Pathways Influence Drosophila Defense Behaviors
Source: Curr Biol. 2016 Apr 4;26(7):981–6. doi: 10.1016/j.cub.2016.02.031 (PMC4826436; doi:10.1016/j.cub.2016.02.031)
Supplement: Document S2. Article plus Supplemental Information [file mmc2.pdf]

Report

Current Biology

Ancient Anxiety Pathways Influence *Drosophila* Defense Behaviors

Graphical Abstract

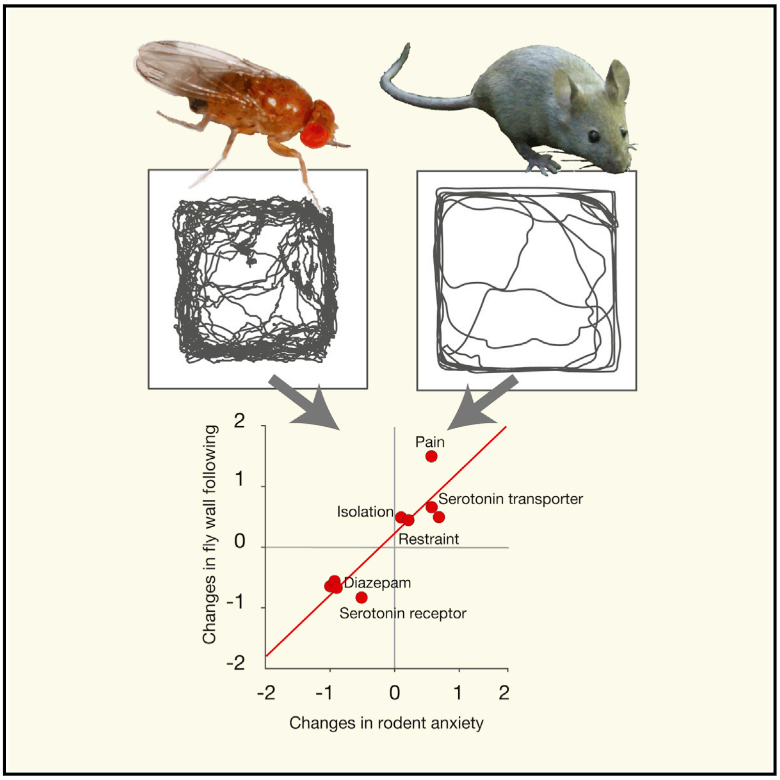

Authors

Farhan Mohammad, Sameer Aryal, Joses Ho, ..., Teng Li Tan, Agnese Eisaka, Adam Claridge-Chang

Correspondence

claridge-chang.adam@duke-nus.edu.sg

In Brief

Mohammad et al. show that orthologs of mammalian anxiety factors govern defense behaviors in the fly and use these behaviors to identify new conserved candidate anxiety genes. Thus, rodent anxiety research may be complemented by *Drosophila* neurogenetic models.

Highlights

- *Drosophila* orthologs of anxiety genes affect fly wall following
- Conserved anxiety genes influence fly defense behaviors similarly to mouse anxiety
- New candidate anxiety genes are identified from fly defense behavior screen
- *Drosophila* identified as a new neurogenetic tool for anxiety research

Mohammad et al., 2016, Current Biology *26*, 981–986
April 4, 2016 ©2016 The Authors
http://dx.doi.org/10.1016/j.cub.2016.02.031

Current Biology
Report

Ancient Anxiety Pathways Influence *Drosophila* Defense Behaviors

Farhan Mohammad,[1,2] Sameer Aryal,[2] Joses Ho,[2] James Charles Stewart,[2] Nurul Ayuni Norman,[2] Teng Li Tan,[2] Agnese Eisaka,[2] and Adam Claridge-Chang[1,2,3,*]
[1]Program in Neuroscience and Behavioral Disorders, Duke-NUS Graduate Medical School, Singapore 138673, Singapore
[2]Institute for Molecular and Cell Biology, Agency for Science Technology and Research, Singapore 138673, Singapore
[3]Department of Physiology, National University of Singapore, Singapore 138673, Singapore
*Correspondence: claridge-chang.adam@duke-nus.edu.sg
http://dx.doi.org/10.1016/j.cub.2016.02.031

SUMMARY

Anxiety helps us anticipate and assess potential danger in ambiguous situations [1–3]; however, the anxiety disorders are the most prevalent class of psychiatric illness [4–6]. Emotional states are shared between humans and other animals [7], as observed by behavioral manifestations [8], physiological responses [9], and gene conservation [10]. Anxiety research makes wide use of three rodent behavioral assays—elevated plus maze, open field, and light/dark box—that present a choice between sheltered and exposed regions [11]. Exposure avoidance in anxiety-related defense behaviors was confirmed to be a correlate of rodent anxiety by treatment with known anxiety-altering agents [12–14] and is now used to characterize anxiety systems. Modeling anxiety with a small neurogenetic animal would further aid the elucidation of its neuronal and molecular bases. *Drosophila* neurogenetics research has elucidated the mechanisms of fundamental behaviors and implicated genes that are often orthologous across species. In an enclosed arena, flies stay close to the walls during spontaneous locomotion [15, 16], a behavior proposed to be related to anxiety [17]. We tested this hypothesis with manipulations of the GABA receptor, serotonin signaling, and stress. The effects of these interventions were strikingly concordant with rodent anxiety, verifying that these behaviors report on an anxiety-like state. Application of this method was able to identify several new fly anxiety genes. The presence of conserved neurogenetic pathways in the insect brain identifies *Drosophila* as an attractive genetic model for the study of anxiety and anxiety-related disorders, complementing existing rodent systems.

RESULTS

Flies Follow the Walls of an Enclosed Chamber

Flies in enclosed chambers spent a large proportion of time near the walls (Figures 1 and S1) [18, 19]. While flies were able to crawl on all surfaces—floor, walls, and ceiling (Figure 1A)—cumulative locomotion traces were strikingly similar to rodent thigmotaxis data from open fields (Figure 1B) [14]. Flies on all surfaces were close to the wall, often 3–4 mm away from the center of a 5-mm chamber (Figure S1C). This behavioral feature, but not locomotion itself, was persistent (Figures S1D–S1F). We termed this behavior ‘‘wall following’’ (WAFO).

Diazepam Reduces Fly Wall Following

Benzodiazepines reduce anxiety by modulating $GABA_A$ receptors [20], and their binding site is evolutionarily conserved [21]. Diazepam reduces anxiety in three important rodent defense behavior assays: the open field (OF), the elevated plus maze (EPM), and the light/dark box [11]. In flies, diazepam had a pronounced effect on fly WAFO at three doses (Figure 1C). Raw behavioral metrics may have an indirect relationship to internal state and are not comparable across diverse experimental systems, for example, between different assays in distinct species. To contextualize the diazepam result, we calculated a standardized effect size (Hedges' *g*) from the diazepam-induced WAFO change (Figure 1C, lower panel) and compared it with a meta-analytic rodent anxiety diazepam effect size calculated from 382 published rodent experiments (http://dx.doi.org/10.1101/020701). Diazepam effect sizes in both systems were comparable (Figure 1D).

Altering *d5-HT1B* Function Has WAFO Effects that Are Concordant with Mouse Anxiety

Genetic experiments in mouse previously demonstrated that deleting and overexpressing the gene for the mammalian 5-HT1A receptor (m5-HT1A) produced moderate effects on rodent anxiety (http://dx.doi.org/10.1101/020701). *Drosophila* has two serotonin class 1 receptor genes with similarity to *m5-HT1A: d5-HT1A* and *d5-HT1B*. The function of these genes was knocked down in adult flies with lines expressing RNAi under the control of a warm-induced pan-neuronal driver, *nSyb-Gal4, tub-Gal80*^{ts} [22, 23]. Alterations of *d5-HT1A* expression with two RNAi lines and one cDNA responder produced only minor changes in WAFO (Figure 2A). However, the use of RNAi and overexpression to alter levels of *d5-HT1B* produced pronounced effects on WAFO (Figure 2B). These *d5-HT1B* effect sizes were of a comparable magnitude to the mouse anxiety effects from *m5-HT1A* lesions (Figure 2D) (http://dx.doi.org/10.1101/020701). Control experiments with warm treatment of control flies had trivial WAFO effects

This is an open access article under the CC BY license (http://creativecommons.org/licenses/by/4.0/).

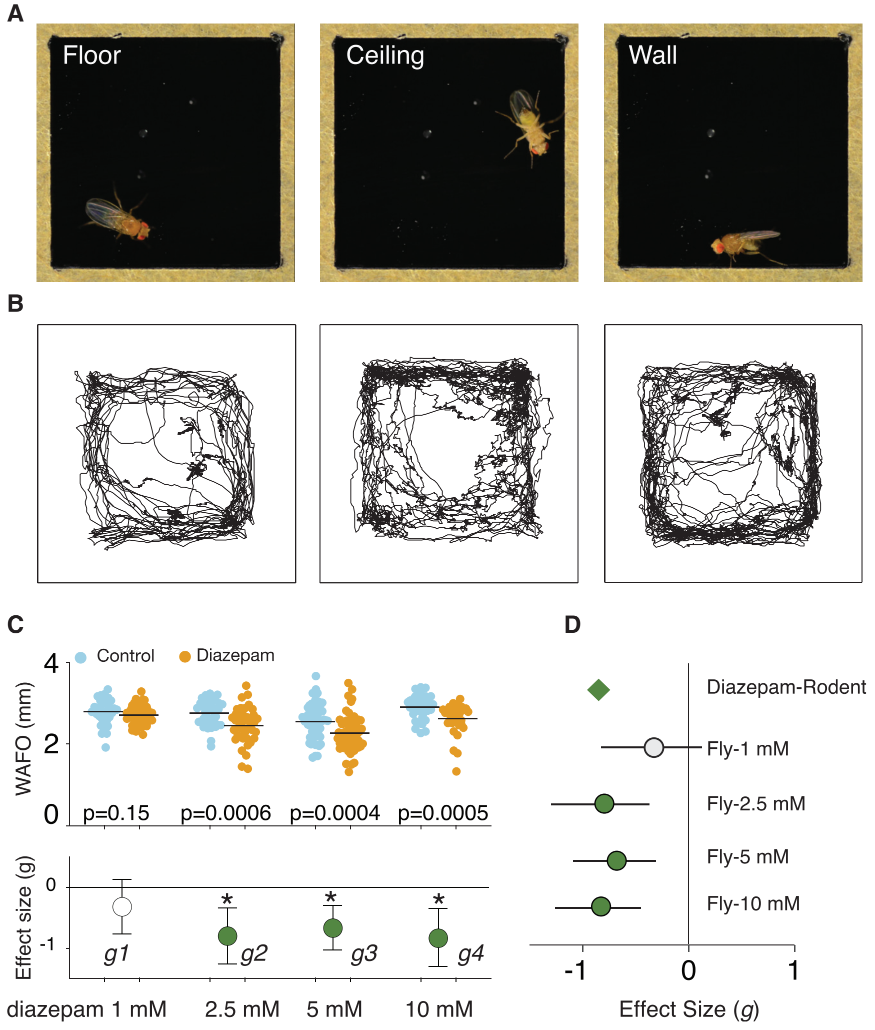

Figure 1. *Drosophila* Wall Following Behavior Is Reduced by Diazepam

(A) Flies in a glass-topped arena walk on all interior surfaces.

(B) Tracking data from a 10-min experiment reveal that flies mainly walk in the perimeter of the arena.

(C) Flies fed with diazepam had decreased WAFO compared with controls ($g1$ = −0.32, $g2$ = −8.0, $g3$ = −0.67, $g4$ = −0.83, n = 40, 40). Fly WAFO was measured as mean distance from center in millimeters. Dots indicate the mean distance from center for individual flies; horizontal line indicates the mean distance from center (mm). p values determined by Mann-Whitney *U*. The lower axis represents the effect size in Hedges' *g* with 95% CI. Green circles and asterisk (*) mark a statistically significant ($p < 0.05$) decrease in behavior.

(D) Standardized mean effect sizes of diazepam effects on rodent anxiety (−0.85 *g* [95 CI −0.74, −0.96]) and fly WAFO (−0.83 *g* [95 CI −0.42, −0.91]) have comparable magnitudes.

See also Figure S1.

(Figures 2A and 2B). We conclude that manipulating *d5-HT1B* function influences fly WAFO in ways that parallel the effects that altering *m5-HT1A* expression has on mouse defense behaviors.

Concordant *SERT* Effects on Fly WAFO and Mouse Anxiety

Deletion of *mSert* produces an increase in mouse anxiety (http://dx.doi.org/10.1101/020701). In flies, reducing *dSerT* mRNA levels with either of two RNAi alleles increased WAFO (Figure 2C). Flies expressing transgenic *dSerT* at 12× elevated levels (Figure S2P) had lowered WAFO (g = −0.53; Figure 2E), echoing the low anxiety observed in mice expressing elevated *mSert* (http://dx.doi.org/10.1101/020701). Control, warm-treated flies underwent no WAFO change (Figure 2C).

Concordant Stress Effects on Fly WAFO and Mouse Anxiety

Environmental stress drives anxiety [24]. Subjecting flies to heat shock stress elicited a large WAFO increase (Figure 3A), concordant with the effect of acute pain on rodent anxiety (Figure S3D) ([25]; http://dx.doi.org/10.1101/020701). Diazepam reduced WAFO in heat-stressed flies, much as it did for flies at 25°C (Figures S2Q and S2R). Physically restraining flies produced a WAFO increase that was concordant with the anxiogenic effect of restraint in rodents (Figure S2E) (http://dx.doi.org/10.1101/020701). Ten days of social isolation stress increased fly WAFO (Figure S2F), an outcome that is concordant with isolation's effect on rodent anxiety (http://dx.doi.org/10.1101/020701). The corticotropin-releasing hormone receptor 1 (CRHR1) is associated with mammalian stress, and knockout mice have lower anxiety; the fly homolog is the diuretic hormone 44 receptor 1 (DH44-R1) [26, 27]. Reducing *Dh44-R1* expression (Figure S2O) reduced WAFO (Figure S2K), consistent with mouse data (http://dx.doi.org/10.1101/020701). Interestingly, *Dh44-R1* mRNA levels were dramatically altered by all three stressors (Figure S4D).

Anxiotropic Manipulations Influence *Drosophila* Light/Dark Choice

A second fly shelter/exposure assay with anxiety concordance would verify that exposure avoidance correlates with fly anxiety. The rodent light/dark choice assay examines light avoidance [12]. We used a simple chamber (Figure S3A) to measure changes in fly light/dark choice in response to anxiety manipulations. Of nine interventions, six (diazepam, *d5-HT1B* loss of function, *dSerT* overexpression, heat, restraint, and social isolation) had substantial statistical effects that were concordant with rodent anxiety data (Figure S3H). The other three were also directionally concordant but had modest, non-statistically significant effects on light/dark choice (*d5-HT1B* overexpression, *dSerT* knockdown, and *Dh44-R1* knockdown). These data largely verify the hypothesis that exposure avoidance measures a fly anxiety state.

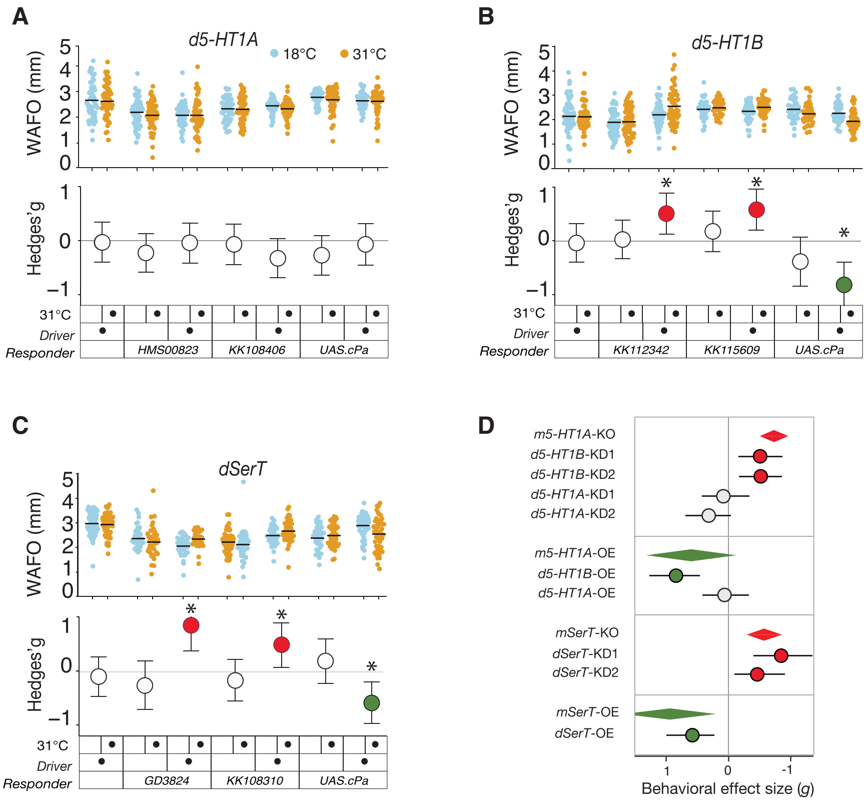

Figure 2. Anxiety-Concordant Effects of Serotonin Gene Lesions on Fly WAFO

(A) Genetic lesions of *d5-HT1A* produced only minor effects in WAFO. Blue dots are untreated flies; orange dots are pre-warmed to 31°C as for GAL80^{ts} derepression. The lower axes show Hedges' g; responder alleles are named in the boxes. The driver is *nSyb-Gal4, Tub-Gal80^{ts}*.

(B) Genetic lesions of *d5-HT1B* had moderate and statistically significant effects on WAFO: knockdown caused increases (*d5-HT1B^{KK112342}* $g = 0.51$, $p = 9 \times 10^{-3}$; *d5-HT1B^{KK115609}* $g = 0.58$, $p = 2 \times 10^{-3}$), while overexpression elicited a decrease ($g = -0.82$, $p = 7.4 \times 10^{-5}$, n = 53, 54). Red and green circles indicate a statistically significant WAFO change.

(C) Knockdowns of *mSerT* with two RNAi lines produced consistent WAFO increases (*SerT^{GD3824}* $g = 0.63$, $p = 8.2 \times 10^{-4}$, n = 60, 55; *SerT^{KK108310}* $g2 = 0.48$, $p = 0.2 \times 10^{-2}$, n = 60, 40), and overexpression decreased WAFO (*dSerT$^{Scer\backslash UAS.cPa}$* $g = -0.53$, $p = 1.8 \times 10^{-3}$, n = 73, 75). Warm-treated controls for *d5-HT1A*, *d5-HT1B*, and *mSerT* UAS transgenes underwent modest, non-statistically significant changes.

(D) A comparison of mouse anxiety gene effect sizes and fly ortholog WAFO effect sizes indicates they are concordant in direction and magnitude, except for *d5-HT1A* knockdowns. Diamonds indicate averaged meta-analytic values; circles indicate fly WAFO effect; lateral vertices and error lines are 95% CI.

See also Figure S2.

Effects in *Drosophila* WAFO and Light/Dark Choice Are Predictive of Rodent Anxiety Effects

Fly and rodent effect sizes for all interventions were subjected to cross-species linear regression. The regression models indicated that fly ΔWAFO data are largely predictive of rodent anxiety changes (R^2_{adj} = 0.77 95% confidence interval [95 CI 0.47, 0.75]), as are the fly Δlight/dark choice outcomes (R^2_{adj} = 0.81 [95 CI 0.58, 0.82]; Figures 3A and 3D). These results are compatible with the hypothesis that fly WAFO and fly light/dark choice, like rodent anxiety assays, test an anxiety-related brain state.

Fly Defense Behaviors Are Distinct from Motor Activity

Motor activity and anxiety behavior are related phenotypes. Tranquilizers like diazepam also have sedative effects, and such overlap might also apply to neurogenetic systems. If WAFO and/or light/dark choice changes were purely a result of speed changes, this would erode confidence in their specificity to anxiety. However, this was not the case. Walking speed was altered in ways that were dissociated from WAFO (Figures S1F, S2A–S2C, and S2H–S2K). Individual flies' WAFO metrics were poorly correlated with "raw" walking speed (WAFO-locomotion R^2_{adj} = 0.18 [95 CI 0.17, 0.19], $p = 1.0 \times 10^{-91}$, n = 2,046), as were their light/dark preferences (shade preference-locomotion R^2_{adj} = 0.05 [95 CI 0.04, 0.06], $p = 1.0 \times 10^{-13}$, n = 1,138). Additional regression analyses of fly walking speed changes (Δspeed) indicated that these could explain less than four-tenths of WAFO change variance (ΔWAFO; Figure 3C) and only a tenth of Δlight/dark variance (Figure 3F). Cross-species analyses indicated that fly speed changes were weakly predictive of rodent anxiety: only a fifth (WAFO; Figure 3B) and 6% (light/dark; Figure 3E) of variance was explained. Thus, while locomotor changes contribute to ΔWAFO and Δlight/dark choice, they are not the main driver.

Identification of *5-HT2B, tsr, tmod, CCKLR-17D1*, and *CCKLR-17D3* as Fly Anxiety Factors

Wall following assays were used to identify fly anxiety gene candidates. Systematic review found that serotonin class 2 receptor knockouts have not been tested for their mouse anxiety role (http://dx.doi.org/10.1101/020701). Functional alterations of the two fly class 2 receptor genes, *d5-HT2A* and *d5-HT2B*, found that only the latter had consistent, substantial effects on WAFO (Figure 4B). Fly orthologs of candidate anxiety genes found at quantitative trait loci (QTLs) identified from a mouse genetic experiment were screened [28]. Of 17 genes, four showed WAFO alterations: *twinstar* (*tsr*), two *Cholecystokinin-like receptor* genes (*CCKLR-17D3* and *CCKLR-17D1*), and *tropomodulin* (*tmod*) (Figure 4), which are homologs of mouse *cofilin 1* (*Cfl1*), *cholecystokinin B receptor* (*Cckbr*), and *Tropomodulin-2* (*Tmod2*), respectively. Control tests of 17 randomly selected orthologs found none produced WAFO effects (Figure 4D). Interestingly, two mouse orthologs of the four fly anxiety candidate genes are known to anxiety research. *Cofilin-1* is a mouse anxiety gene with a knockout having a concordant outcome to the fly WAFO result [28]. Mouse *Cckbr* codes for cholecystokinin receptor, and its deletion has an effect concordant with knockdown effects of fly WAFO [29].

CellPress

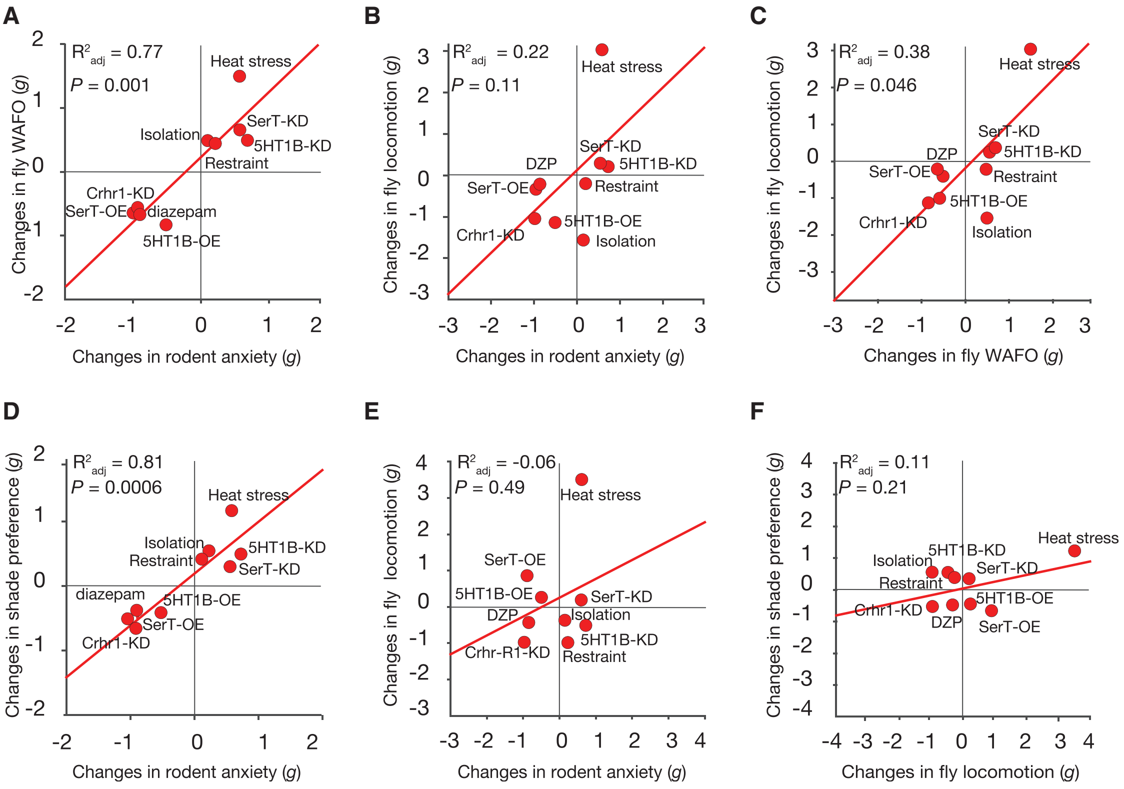

Figure 3. Fly Defense Behavior Outcomes Are Concordant with Anxiety Outcomes

(A) A strong correlation between rodent anxiety and fly WAFO data for nine comparable manipulations (R^2_{adj} = 0.77 [95 CI 0.58, 0.83]). The horizontal axis shows rodent meta-analytic *g* values; the vertical axis displays fly WAFO *g* values. The red line is the least-squares fit; p is for the F statistic of the model.
(B) Walking speed changes in the square arena are weakly correlated with rodent anxiety outcomes (R^2_{adj} = 0.22 [95 CI 0.0, 0.30]).
(C) WAFO is moderately related to locomotion in the square arena (R^2_{adj} = 0.38 [95 CI 0.06, 0.49]).
(D) Light/dark choice outcomes are strongly correlated with rodent effect sizes (R^2_{adj} = 0.81 [95 CI 0.64, 0.86]).
(E) Changes in locomotion in the light/dark arena are weakly correlated with rodent anxiety outcomes (R^2_{adj} = 0.06 [95 CI 0.0, 0.09]).
(F) Light/dark choice outcomes are poorly correlated with locomotion (R^2_{adj} = 0.11 [95 CI 0.0, 0.14]).
See also Figure S3.

DISCUSSION

The results verify the hypothesis that exposure avoidance behaviors of *Drosophila* share underlying neurogenetic pathways with mammalian anxiety. A GABA-modulating drug, serotonin receptor and transporter alterations, a stress peptide receptor, and environmental stressors produced effects that were concordant with comparable manipulations in mammalian anxiety-related behaviors. A regression comparison of fly behavior data and rodent anxiety data indicated that the two are similar. The high coefficients of determination observed in the interspecies comparisons are remarkable in that they would not be expected to account for sources of variance that include sampling error, within- and between-lab heterogeneity, publication bias, >600 million years of evolutionary divergence, or the difference between semi-acute knockdowns and lifelong knockouts.

A candidate survey newly implicated *d5-HT2B*, *tsr*, *tmod*, *CCKLR-17D3*, and *CCKLR-17D1* in fly anxiety. The anxiolytic effect of *tsr* supports the hypothesis that actin microfilament stability is connected to anxiety [28], consistent with ideas that actin polymerization influences anxiety via aversive memory formation and stability [30] and/or related processes [31]. Similarly, that CCK-like receptor knockdowns reduce fly anxiety supports the hypothesis that CCK receptors are involved in anxiety and fear [32, 33], with a role proposed specifically for the mammalian cholecystokinin B receptor (CCKBR) [29]. In flies, the putative ligand for the CCKLR receptors is DROSULFAKININ (DSK); intriguingly, *CCKLR-17D1* and *dsk* mutants have deficits in a larval stress-induced escape behavior [34]. The implication of CCK-like receptors in fly defense behaviors suggests that this is an anxiety-related signaling system, like GABA, serotonin, and Dh44-R1/CRHR1. Most of the orthologous gene knockdowns produced no WAFO effect, suggesting that the QTL hits include false positives and that WAFO genes are relatively rare.

Anxiety research has struggled to find new therapeutics [11]. Bringing the neurogenetic tools and larger sample sizes of *Drosophila* to bear on anxiety promises to complement rodent model analysis of anxiety and anxiety disorders.

SUPPLEMENTAL INFORMATION

Supplemental Information includes Supplemental Experimental Procedures and four figures and can be found with this article online at http://dx.doi.org/10.1016/j.cub.2016.02.031.

CellPress

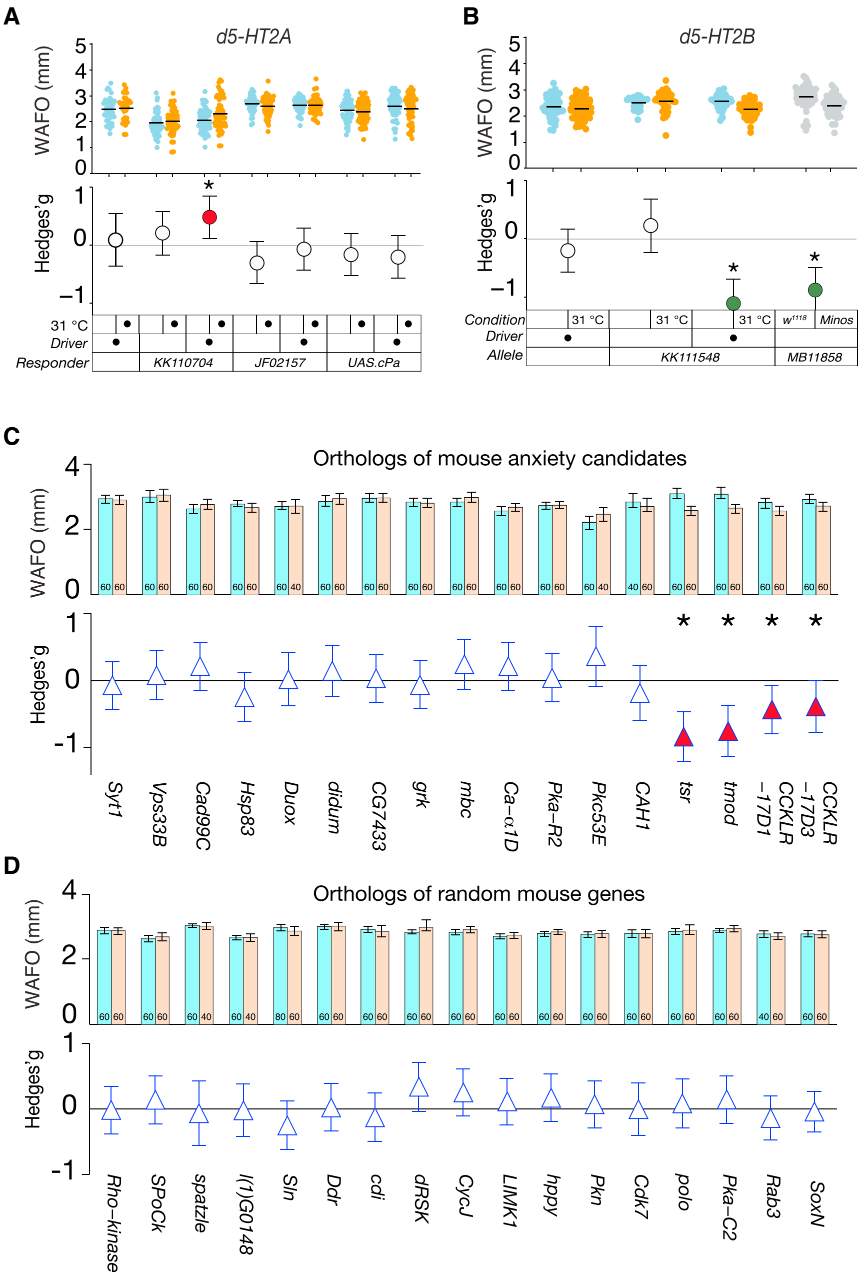

Figure 4. Identification of Candidate Fly Anxiety Genes

(A) RNAi knockdown with *d5-HT2A*^{KK110704} increased WAFO (g = 0.48, p = 1 × 10^{-2}), but this effect was not confirmed by a second RNAi allele (*d5-HT2A*^{JF02157} g = −0.07, p = 6.9 × 10^{-1}) or overexpression (*d5-HT2A*$^{Scer\setminus UAS.cPa}$ g = −0.21, p = 0.28). Warm-treated controls underwent non-statistical WAFO alterations.

(B) Knockdown of *d5-HT2B* with *d5-HT2B*^{KK111548} produced a decrease in WAFO (g = −1.1, p = 6.8 × 10^{-08}) as did a *Minos* transposon insertion into the gene: *d-HT2B*^{MB11858} (g = −0.88, p = 4.1 × 10^{-06}).

(C) Orthologs of candidate mouse anxiety genes were knocked down in the adult fly and tested for WAFO changes. Four knockdowns produced statistically significant reductions in WAFO: *tsr*^{KK108706} (g = −0.89, p = 5.0 × 10^{-6}); *tmod*^{KK108701} (g = −0.81, p = 1.8 × 10^{-5}); *CCKLR−17D1*^{KK108482} (g = −0.45, p = 3.5 × 10^{-4}); and *CCKLR−17D3*^{KK110484} (g = −0.40, p = 1.2 × 10^{-2}). Sample sizes are indicated at the base of the bars.

(D) Seventeen randomly selected orthologs' knockdowns had trivial effects on WAFO.

See also Figure S4.

AUTHOR CONTRIBUTIONS

Conceptualization, F.M. and A.C.-C.; Methodology, F.M. and A.C.-C.; Software, F.M., S.A., J.H., J.C.S., and A.C.-C.; Investigation, F.M., T.L.T., N.A.N., and A.E.; Writing, F.M. and A.C.-C.; Visualization, F.M.; Supervision, A.C.-C.; Project Administration, A.C.-C.; Funding Acquisition, A.C.-C.

ACKNOWLEDGMENTS

We thank Jonathan Flint, Gero Miesenböck, Justin Blau, Ajay Mathuru, and members of the A.C.-C. lab for their comments. We thank Helen Whitley, Martin Goodson, and Shankar Chandrasekhar Iyer for technical assistance. The authors were supported by Biomedical Research Council block grants to the Neuroscience Research Partnership and the Institute of Molecular and Cell Biology. F.M. and A.C.-C. received support from Duke-NUS Graduate Medical School. A.C.-C. received support from a Nuffield Department of Medicine Fellowship, a Wellcome Trust block grant to the University of Oxford, and NARSAD Young Investigator Award 17741. J.H., S.A., and A.E. were supported by awards from the A*STAR Graduate Academy.

Received: December 10, 2014
Revised: December 13, 2015
Accepted: February 10, 2016
Published: March 24, 2016

REFERENCES

1. Blanchard, D.C., Griebel, G., and Blanchard, R.J. (2001). Mouse defensive behaviors: pharmacological and behavioral assays for anxiety and panic. Neurosci. Biobehav. Rev. *25*, 205–218.

2. McNaughton, N., and Corr, P.J. (2004). A two-dimensional neuropsychology of defense: fear/anxiety and defensive distance. Neurosci. Biobehav. Rev. *28*, 285–305.
3. Belzung, C., and Philippot, P. (2007). Anxiety from a phylogenetic perspective: is there a qualitative difference between human and animal anxiety? Neural Plast. *2007*, 59676.
4. Kessler, R.C., Heeringa, S., Lakoma, M.D., Petukhova, M., Rupp, A.E., Schoenbaum, M., Wang, P.S., and Zaslavsky, A.M. (2008). Individual and societal effects of mental disorders on earnings in the United States: results from the national comorbidity survey replication. Am. J. Psychiatry *165*, 703–711.
5. DiLuca, M., and Olesen, J. (2014). The cost of brain diseases: a burden or a challenge? Neuron *82*, 1205–1208.
6. Baldwin, D.S., Anderson, I.M., Nutt, D.J., Allgulander, C., Bandelow, B., den Boer, J.A., Christmas, D.M., Davies, S., Fineberg, N., Lidbetter, N., et al. (2014). Evidence-based pharmacological treatment of anxiety disorders, post-traumatic stress disorder and obsessive-compulsive disorder: a revision of the 2005 guidelines from the British Association for Psychopharmacology. J. Psychopharmacol. (Oxford) *28*, 403–439.
7. Anderson, D.J., and Adolphs, R. (2014). A framework for studying emotions across species. Cell *157*, 187–200.
8. Darwin, C. (1998). The Expression of the Emotions in Man and Animals (Project Gutenberg).
9. Cannon, W.B. (2005). Bodily Changes in Pain, Hunger, Fear and Rage (Internet Archive).
10. Saudou, F., Boschert, U., Amlaiky, N., Plassat, J.L., and Hen, R. (1992). A family of Drosophila serotonin receptors with distinct intracellular signalling properties and expression patterns. EMBO J. *11*, 7–17.
11. Griebel, G., and Holmes, A. (2013). 50 years of hurdles and hope in anxiolytic drug discovery. Nat. Rev. Drug Discov. *12*, 667–687.
12. Crawley, J., and Goodwin, F.K. (1980). Preliminary report of a simple animal behavior model for the anxiolytic effects of benzodiazepines. Pharmacol. Biochem. Behav. *13*, 167–170.
13. Pellow, S., Chopin, P., File, S.E., and Briley, M. (1985). Validation of open:closed arm entries in an elevated plus-maze as a measure of anxiety in the rat. J. Neurosci. Methods *14*, 149–167.
14. Simon, P., Dupuis, R., and Costentin, J. (1994). Thigmotaxis as an index of anxiety in mice. Influence of dopaminergic transmissions. Behav. Brain Res. *61*, 59–64.
15. Besson, M., and Martin, J.-R. (2005). Centrophobism/thigmotaxis, a new role for the mushroom bodies in *Drosophila*. J. Neurobiol. *62*, 386–396.
16. Lima, S.Q., and Miesenböck, G. (2005). Remote control of behavior through genetically targeted photostimulation of neurons. Cell *121*, 141–152.
17. Iliadi, K.G. (2009). The genetic basis of emotional behavior: has the time come for a *Drosophila* model? J. Neurogenet. *23*, 136–146.
18. Martin, J.R., Ernst, R., and Heisenberg, M. (1999). Temporal pattern of locomotor activity in Drosophila melanogaster. J. Comp. Physiol. A Neuroethol. Sens. Neural Behav. Physiol. *184*, 73–84.
19. Soibam, B., Mann, M., Liu, L., Tran, J., Lobaina, M., Kang, Y.Y., Gunaratne, G.H., Pletcher, S., and Roman, G. (2012). Open-field arena boundary is a primary object of exploration for *Drosophila*. Brain Behav. *2*, 97–108.
20. Sigel, E., and Baur, R. (1988). Allosteric modulation by benzodiazepine receptor ligands of the GABAA receptor channel expressed in Xenopus oocytes. J. Neurosci. *8*, 289–295.
21. Robinson, T., MacAllan, D., Lunt, G., and Battersby, M. (1986). gamma-Aminobutyric acid receptor complex of insect CNS: characterization of a benzodiazepine binding site. J. Neurochem. *47*, 1955–1962.
22. Dietzl, G., Chen, D., Schnorrer, F., Su, K.-C., Barinova, Y., Fellner, M., Gasser, B., Kinsey, K., Oppel, S., Scheiblauer, S., et al. (2007). A genome-wide transgenic RNAi library for conditional gene inactivation in Drosophila. Nature *448*, 151–156.
23. McGuire, S.E., Le, P.T., Osborn, A.J., Matsumoto, K., and Davis, R.L. (2003). Spatiotemporal rescue of memory dysfunction in *Drosophila*. Science *302*, 1765–1768.
24. van Praag, H.M. (2004). Can stress cause depression? Prog. Neuropsychopharmacol. Biol. Psychiatry *28*, 891–907.
25. Marsch, R., Foeller, E., Rammes, G., Bunck, M., Kössl, M., Holsboer, F., Zieglgänsberger, W., Landgraf, R., Lutz, B., and Wotjak, C.T. (2007). Reduced anxiety, conditioned fear, and hippocampal long-term potentiation in transient receptor potential vanilloid type 1 receptor-deficient mice. J. Neurosci. *27*, 832–839.
26. Johnson, E.C., Bohn, L.M., and Taghert, P.H. (2004). Drosophila CG8422 encodes a functional diuretic hormone receptor. J. Exp. Biol. *207*, 743–748.
27. Chang, C.L., and Hsu, S.Y.T. (2004). Ancient evolution of stress-regulating peptides in vertebrates. Peptides *25*, 1681–1688.
28. Goodson, M., Rust, M.B., Witke, W., Bannerman, D., Mott, R., Ponting, C.P., and Flint, J. (2012). Cofilin-1: a modulator of anxiety in mice. PLoS Genet. *8*, e1002970.
29. Zwanzger, P., Domschke, K., and Bradwejn, J. (2012). Neuronal network of panic disorder: the role of the neuropeptide cholecystokinin. Depress. Anxiety *29*, 762–774.
30. Lamprecht, R. (2011). The roles of the actin cytoskeleton in fear memory formation. Front. Behav. Neurosci. *5*, 39.
31. Soetanto, A., Wilson, R.S., Talbot, K., Un, A., Schneider, J.A., Sobiesk, M., Kelly, J., Leurgans, S., Bennett, D.A., and Arnold, S.E. (2010). Association of anxiety and depression with microtubule-associated protein 2- and synaptopodin-immunolabeled dendrite and spine densities in hippocampal CA3 of older humans. Arch. Gen. Psychiatry *67*, 448–457.
32. de Montigny, C. (1989). Cholecystokinin tetrapeptide induces panic-like attacks in healthy volunteers. Preliminary findings. Arch. Gen. Psychiatry *46*, 511–517.
33. Bradwejn, J., Koszycki, D., and Shriqui, C. (1991). Enhanced sensitivity to cholecystokinin tetrapeptide in panic disorder. Clinical and behavioral findings. Arch. Gen. Psychiatry *48*, 603–610.
34. Chen, X., Peterson, J., Nachman, R.J., and Ganetzky, B. (2012). Drosulfakinin activates CCKLR-17D1 and promotes larval locomotion and escape response in *Drosophila*. Fly (Austin) *6*, 290–297.

Current Biology, Volume *26*

Supplemental Information

Ancient Anxiety Pathways Influence *Drosophila* Defense Behaviors

Farhan Mohammad, Sameer Aryal, Joses Ho, James Charles Stewart, Nurul Ayuni Norman, Teng Li Tan, Agnese Eisaka, and Adam Claridge-Chang

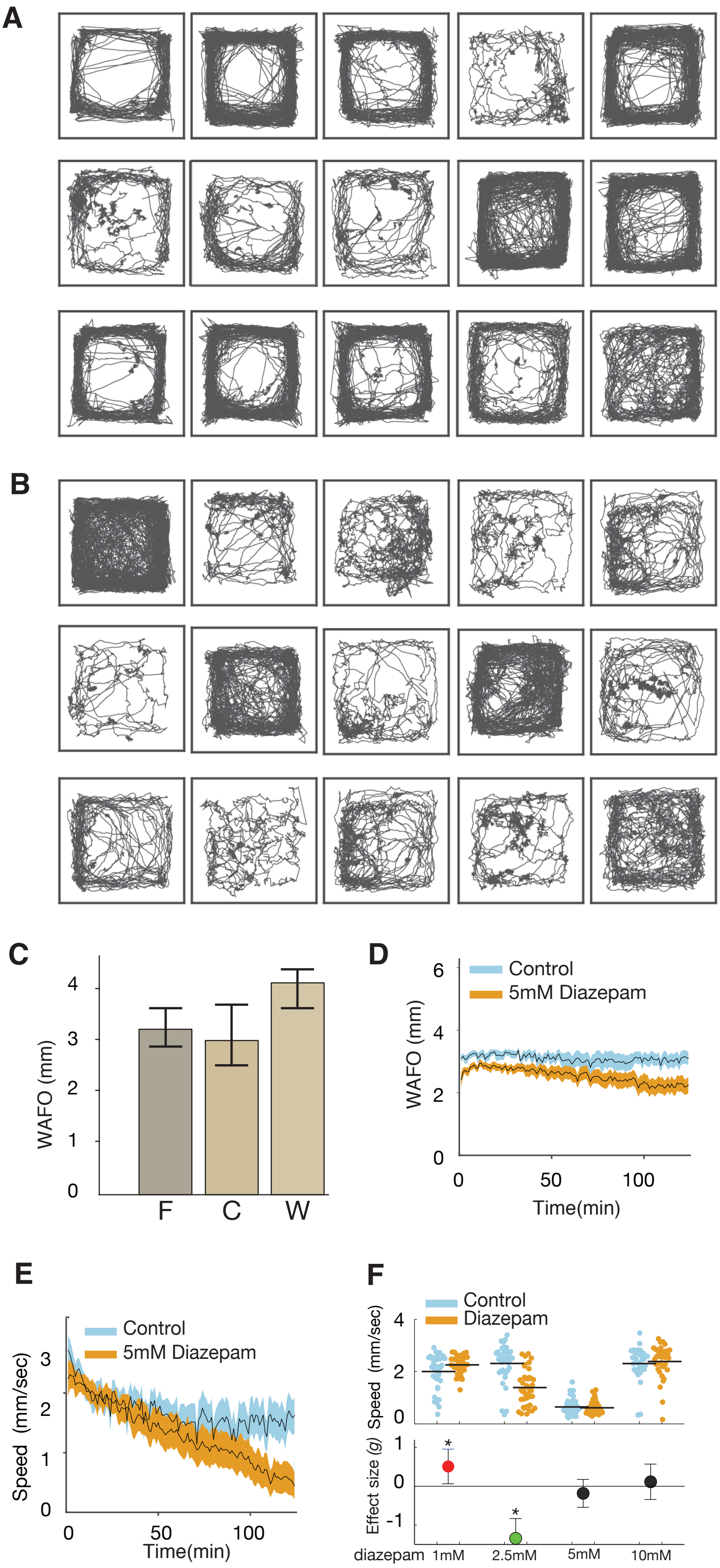

A
B
C
WAFO (mm)
0
1
2
3
4
F
C
W
D
Control
5mM Diazepam
WAFO (mm)
6
4
2
0
0
50
100
Time(min)
E
Control
5mM Diazepam
Speed (mm/sec)
3
2
1
0
0
50
100
Time(min)
F
Control
Diazepam
Speed (mm/sec)
4
2
0
Effect size (g)
1
0
-1
*
*
diazepam 1mM
2.5mM
5mM
10mM

Figure S1

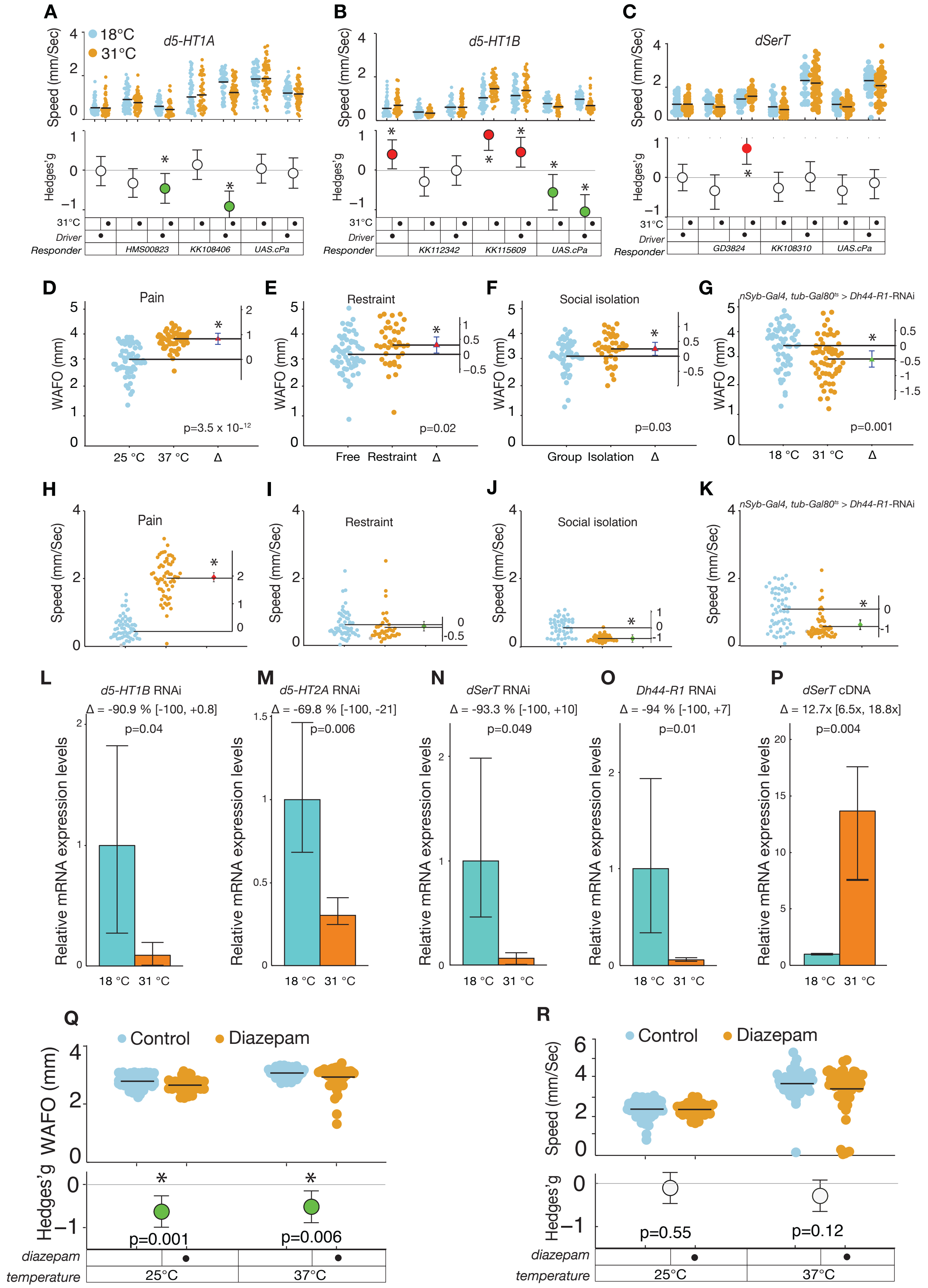

A
18°C
31°C
d5-HT1A
Speed (mm/Sec)
Hedges'g
31°C
Driver
Responder
HMS00823
KK108406
UAS.cPa
B
d5-HT1B
KK112342
KK115609
C
dSerT
GD3824
KK108310
D
Pain
WAFO (mm)
p=3.5 x 10-12
25 °C
37 °C
Δ
E
Restraint
p=0.02
Free
F
Social isolation
p=0.03
Group
Isolation
G
nSyb-Gal4, tub-Gal80ts > Dh44-R1-RNAi
p=0.001
18 °C
31 °C
H
I
J
K
L
d5-HT1B RNAi
Δ = -90.9 % [-100, +0.8]
p=0.04
Relative mRNA expression levels
M
d5-HT2A RNAi
Δ = -69.8 % [-100, -21]
p=0.006
N
dSerT RNAi
Δ = -93.3 % [-100, +10]
p=0.049
O
Dh44-R1 RNAi
Δ = -94 % [-100, +7]
p=0.01
P
dSerT cDNA
Δ = 12.7x [6.5x, 18.8x]
p=0.004
Q
Control
Diazepam
p=0.001
p=0.006
diazepam
temperature
25°C
37°C
R
p=0.55
p=0.12

Figure S2.

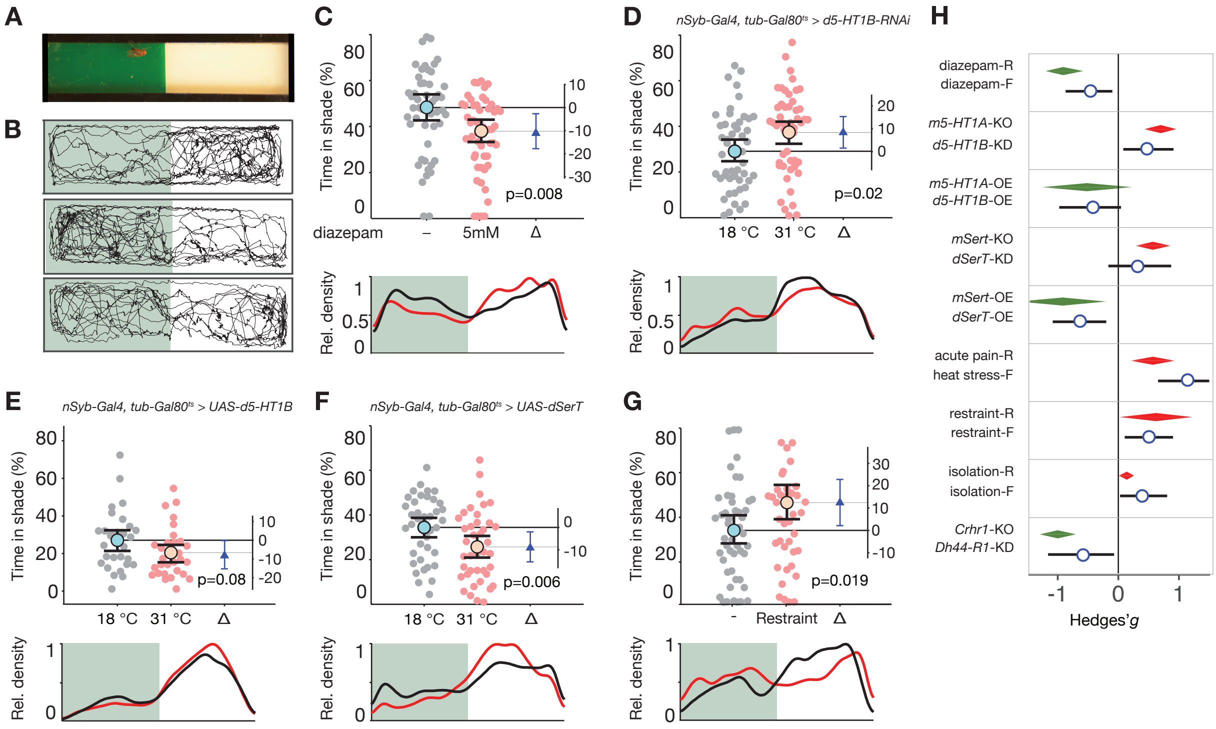
A
B
C
D
nSyb-Gal4, tub-Gal80ts > d5-HT1B-RNAi
H
Time in shade (%)
diazepam
–
5mM
Δ
p=0.008
18 °C
31 °C
p=0.02
Rel. density
E
nSyb-Gal4, tub-Gal80ts > UAS-d5-HT1B
F
nSyb-Gal4, tub-Gal80ts > UAS-dSerT
G
p=0.08
p=0.006
p=0.019
-
Restraint
diazepam-R
diazepam-F
m5-HT1A-KO
d5-HT1B-KD
m5-HT1A-OE
d5-HT1B-OE
mSert-KO
dSerT-KD
mSert-OE
dSerT-OE
acute pain-R
heat stress-F
restraint-R
restraint-F
isolation-R
isolation-F
Crhr1-KO
Dh44-R1-KD
Hedges'g

Figure S3.

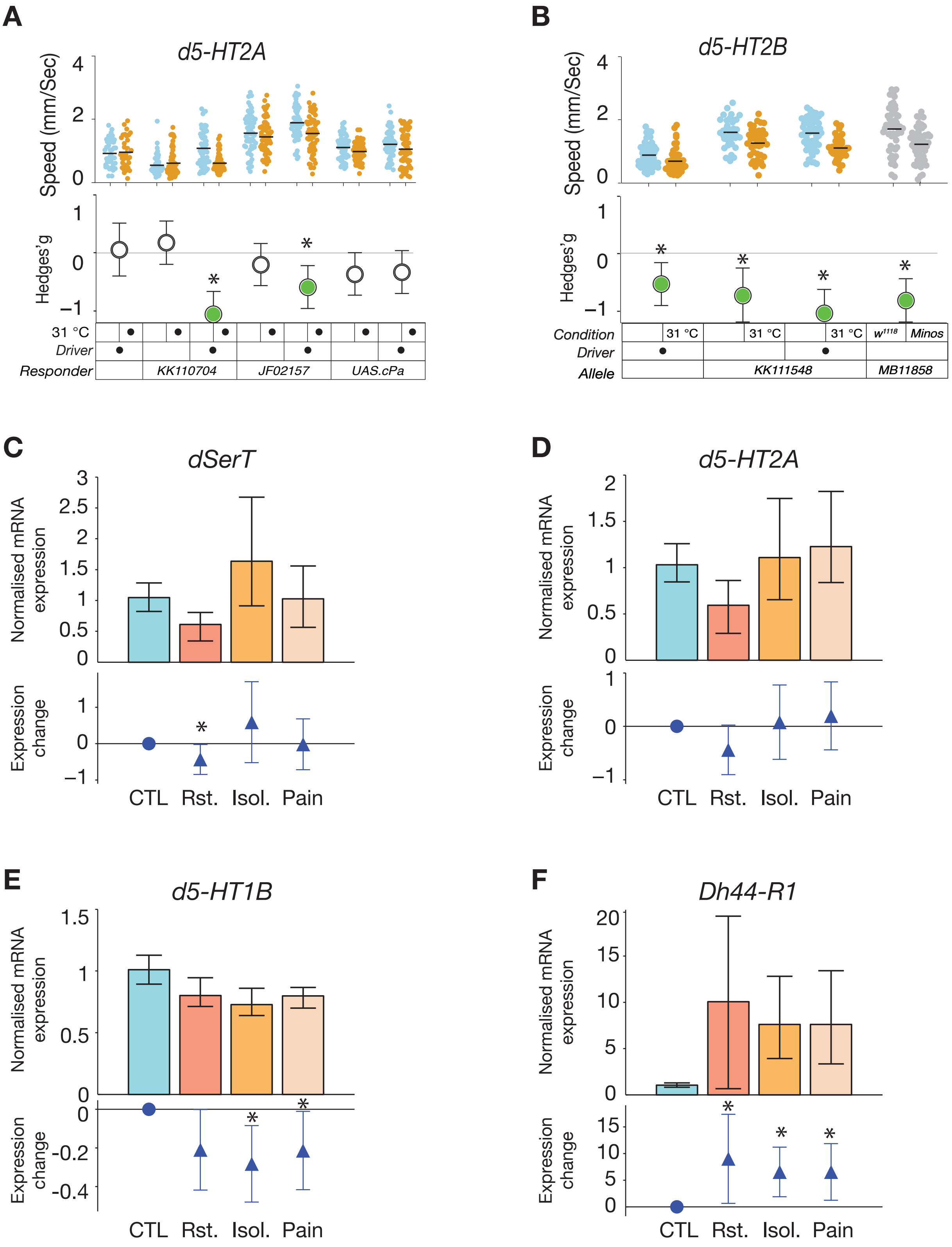
A
d5-HT2A
Speed (mm/Sec)
Hedges'g
31 °C
Driver
Responder
KK110704
JF02157
UAS.cPa
B
d5-HT2B
Condition
Allele
KK111548
MB11858
Minos
C
dSerT
Normalised mRNA expression
Expression change
CTL
Rst.
Isol.
Pain
D
d5-HT2A
E
d5-HT1B
F
Dh44-R1

Figure S4.

SUPPLEMENTAL INFORMATION

Supplemental Figure Legends

Figure S1. Locomotion data for flies treated with diazepam

Refers to Figure 1.

A. Example cumulative traces of the movement of 15 flies treated with solvent.

B. Traces of locomotor behavior of flies treated with diazepam (2.5 mM)

C. When their feet are contacting the floor (F), the ceiling (C), or walls (W) flies stay 4.1 mm [95CI 4.5, 3.6], 3.2 mm [95CI 3.6, 2.8], and 3.0 mm [95CI 3.6, 2.3] from the center, respectively.

D. Both diazepam treated and non-treated flies' WAFO (mean distance from the center) did not change appreciably over 120 min (N = 40 flies). Color shading is 95% confidence intervals.

E. Both diazepam treated and non-treated flies' walking speed slowed over time (N=40 flies). Statistical differences were observed after ~90 minutes.

F. Some diazepam-fed flies have large differences in walking speed; in these experiments, locomotion was statistically increased or decreased in flies fed 1 mM or 2.5 mM, respectively, but this was not conventionally dose-dependent; higher concentrations did not elicit statistical changes.

Figure S2. The effects of receptor gene lesions and stress on WAFO and locomotion

Refers to Figure 2.

A. Knockdown of the *d5-HT1A* receptor with RNAi reduces locomotion, but overexpression has a trivial effect.

B. Locomotion is statistically altered in five experiments with the *d5-HT1B* receptor allele, but these are not attributable to gene knockdown effects as changes are seen in driver-transgene-only control flies subjected to warm treatment.

C. Locomotion is statistically altered in only one *dSerT* experiment.

D. Exposing flies to 37°C dramatically increased proximity to the wall; $g = 1.4$, $P = 3.5 \times 10^{-12}$. The P values in this figure were calculated using the Mann–Whitney *U* method.

E. One hour of restraint stress in a nested pipette tip increased WAFO in flies tested after this treatment, compared with control animals allowed to move freely: $g = 0.41$, $P = 2.4 \times 10^{-2}$, N = 58, 39.

F. Isolating Canton-S male flies from social contact for 10 days led to increased WAFO: $g = 0.475$, P = 0.029, N = 60, 40.

G. Knockdown of *Dh44-R1* with $Dh44\text{-}R1^{KK108591}$ resulted in decreased WAFO: $g = -0.57$, $P = 1.0 \times 10^{-3}$, N = 60, 59.

H. Stressful heat at 37°C has a large effect on walking speed in the square arena (g = 3.1, P = 2.7 × 10^{-19}, N = 60 60).

I. Restraint stress has a negligible effect on walking speed (g = -0.21, P = 4 × 10^{-2}, N = 58, 39).

J. Social isolation suppressed walking speed (g = -1.6, P = 9 × 10^{-9}, N = 49, 40).

K. Knockdown of Dh44-R1 lowers walking speed (g = -1.03, P = 1.2 × 10^{-6}, N = 60, 59).

L. Quantitative PCR of transgenic *Drosophila* lines bearing RNAi or cDNA alleles verifies the effects on mRNA expression. Flies were assayed for mRNA levels after treatment with a protocol identical to the one used for induction prior to behavior. Targeting the *d5−HT1B* receptor gene with $d5-HT1B^{KK112342}$ led to a 91% reduction of the fly head mRNA level (g = −1.2, P = 0.047, N = 5, 5 experiments). The blue bars are normalized expression levels in uninduced flies, the orange bars are data from induced flies. The confidence interval of each percent change was calculated by bootstrap and P was calculated with the Mann-Whitney-Wilcoxon *U* method.

M. Targeting *d5−HT2A* with $d5-HT2A^{KK110704}$ led to a 70% reduction of expression (g = −1.7, P = 0.006, N = 6, 6).

N. Targeting *SerT* with $SerT^{GD3824}$ for three successive nights (~17 hours each) at 31 °C led to a 93% reduction in mRNA (g = −1.2, P = 0.050, N = 3, 3).

O. Induction of RNAi from the $Dh44-R1^{KK108591}$ allele led to a 94% reduction in mRNA (g = −0.84, P = 0.027, N = 4, 5).

P. Induction of the $SerT^{Scer-UAS.cPa}$ transgene led to a 12.7-fold increase in mRNA levels (g = 2.4, P = 0.004, N = 6, 6).

Q. Flies fed with 5 mM diazepam show reduced WAFO at both 25°C (g = −0.63, p = 0.001, N = 60, 60) and stressful 37°C (g = −0.52, p = 0.006, N = 60, 60).

R. Diazepam 5 mM has a minimal effect on locomotion at 25°C (g = −0.11, p = 0.55, N = 60,60) and modest reduction at 37°C (g = −0.29, p = 0.12, N = 60,60).

Figure S3: A light/dark choice assay for *Drosophila* and anxiety manipulations

Refers to Figure 3.

A. A photograph of a light/dark choice chamber in which one half is shaded by a green filter.

B. Representative cumulative traces of flies moving in light/dark chambers for 10 minutes.

C. Starved wild type flies fed 5 mM diazepam displayed decreased time spent on the shaded side (g = -0.52, P = 0.008, N = 52, 55) relative to flies fed the carrier liquid (5% sucrose, 20% v/v Tween-80) alone. The lower panel shows the relative density of fly locations for all video frames from all 107 flies without (black) and with diazepam treatment (red), dual normalized histograms in which the maximal density value is set to 1. Green box indicates the shaded area. All P values were calculated by the Mann–Whitney *U* method.

D. Flies targeted with an RNAi against *d5-HT1B* spent an increased amount of time in the dark shaded area: $g = 0.47$, P = 0.02, N = 50, 50.

E. Flies carrying an induced *d5-HT1B* cDNA transgene showed decreased time spent in the shaded region: $g = -0.449$, P = 0.08, N = 30, 30, not statistically significant

F. Flies with overexpressed *dSerT* decreased the time they spent in the shaded region: $g = -0.62$, P = 0.0067, N = 40, 40.

G. Exposing Canton-S flies to one hour of restraint stress increased time spent in the shaded region: $g = 0.49$, P = 0.019, N = 50, 46.

H. Comparison of effect sizes from meta-analyses of published rodent data (diamonds) with corresponding fly light/dark assay results (blue). The fly primary data are compatible with the rodent meta-analytic data, except for isolation (which has the same direction, but is substantially smaller in fly).

Figure S4: The effects of serotonin class 2 receptor gene lesions on locomotion and stress-related anxiety gene expression.

Refers to Figure 4.

A. The *d5-HT2A* knockdowns reduced walking speed, but overexpression had little effect.

B. Reducing function of *d5-HT2B* lowered walking speed in all experiments, but this could not be attributed to the gene function as controls were also affected.

C. Serotonin transporter (*dSerT*) mRNA levels were statistically unchanged by diverse stressors: restraint Rst., P = 0.11, N = 6, 6; isolation Isol., P = 0.34, N = 6, 6; heat shock at 37°C (P = 0.10, N = 6, 5). Flies were subjected to one of three stressors prior to being assayed by qPCR for expression levels relative to untreated flies (CTL). For each experiment, the top panel indicates expression relative to control flies and the bottom panel plots the expression fold change and its 95% confidence intervals. Confidence interval bars of the contrast (change) indicate statistical significance when they do not cross the zero line (this is also indicated by the red asterisks). All P values in this figure were calculated using the Mann–Whitney–Wilcoxon *U* method.

D. *d5-HT2A* mRNA remained statistically unchanged in response to any of the stressors: restraint (FC = -0.44, p = 0.88,, N = 6, 4), isolation (= 0.0.08, p = 0.1, N = 6, 6), heat (*FC* = 0.19, p = 0.71, N = 6, 5).

E. *d5-HT1B* mRNA in fly heads is downregulated in response to all three stressors. Exposing flies to one hour of restraint stress reduced *d5-HT1B* mRNA levels (−0.21 × decrease, P = 0.14, N = 6, 5), although this was not statistically significant. Housing male flies in isolation lowered *d5-HT1B* levels (−0.28 × decrease, P = 0.016, N = 6, 6). Exposing flies to 37°C for 10 minutes also decreased *d5-HT1B* mRNA (−0.21 × decrease, P = 0.01, N = 6, 4).

F. *Dh44-R1* mRNA was dramatically elevated by all three stressors: restraint (9 × increase [95CI 3, 12]; N = 6, 4; P = 0.01), isolation (6.6 × increase [95CI 3, 11]; N = 6, 4; P = 0.01) and stressful heat (6.5 × increase [95CI 2, 19]; N = 6, 3; P = 0.03).

Supplemental experimental procedures

Fly strains

Flies were cultured and maintained on fly medium at 24°C and 60% humidity on a 12 h light: 12 h dark cycle unless otherwise mentioned. Wild-type stocks were *w*^{1118} and Canton-Special (C-S). Strains carrying inverted repeat RNAi transgenes were obtained from the Vienna Drosophila Research Centre (VDRC) and Bloomington Drosophila Stock Center (BDSC). The *UAS-RNAi* lines used were: *d5-HT1A*^{KK108407}, *d5-HT1A*^{HMS00823}, *d5-HT1B*^{KK112342}(v109929), *d5-HT1B*^{KK115609}(v110128), *d5-HT2A*^{KK110704} (v102105), *d5-HT2A*(JF02157), *d5-HT2B*^{KK111548} (v102356), *d-HT2B*^{MB11858}, *SerT*^{KK108310} (v100584), *SerT*^{GD3824} (v11346), and *Dh44-R1*^{KK108591} (v110708). UAS lines were obtained from the Bloomington Drosophila Stock Center (BDSC): *SerT*$^{Scer\backslash UAS.cPa}$ [S1], *d5-HT1A*$^{Scer\backslash UAS.cPa}$ and *d5-HT1B*$^{Scer\backslash UAS.cPa}$ [S2], and 5-HT2$^{Scer\backslash UAS.cPa}$ [S3]. *nSyb-Gal4, Tub-Gal80*^{ts} conditional pan-neural driver strain was generated by standard methods from the constituent transgenics: *nSyb-GAL4* was a gift from Bassem Hassan and *Tub-Gal80*^{ts} was acquired from BDSC.

Transgenic animal preparation

For temperature-controlled transgene expression [S4], *nSyb-Gal4, tub-Gal80*^{ts}*/TM3* virgin females were crossed with males carrying a responder transgene (*i.e. UAS-RNAi* or *UAS-cDNA)* [S5]. This combination allowed temporal control of neural expression using thermal de-repression of GAL80^{ts} inhibition of GAL4-activated transcription at 31°C. We used comparisons between isogenic fly groups so as to avoid possible effects from strain genetic background and temporal control to exclude developmental effects. The progeny of these crosses were raised at 18°C to maintain repression of the responder gene. Newly eclosed non-*TM3* males were isolated and maintained as a group of 24 flies in vials (2.4 cm diameter × 9.4 cm height) for 2–3 days before the behavioral assays. Vials of these animals were either maintained at 18°C until assaying, or treated to one or more overnight (~16 h) interval/s at 31°C. A single night of induction was initially used for each responder. In the case of *dSerT*^{GD3824}, no behavioral effect was seen with 1 night induction so the experiment was repeated with 3 consecutive nights at 31°C, with days at 18°C. Prior to the behavioral assay, flies were held for a 2–3 h recovery period at 25°C. In addition to the non-induced flies carrying *UAS* transgenes, further control animals were generated by crossing the driver line with wild type flies (either C-S or a *w*^{1118} strain received from VDRC) and raising the progeny under identical regimes as the flies carrying the responder transgenes. *d-HT2B*^{MB11858} line was first outcrossed to W1118 background for 6 consecutive generations; control and experimental flies were raised together at identical conditions before assaying them for WAFO at 25°C.

Candidate anxiety gene screen

The following VDRC RNAi alleles were used to assess the orthologs of the candidate anxiety genes from the mouse QTL study: *Syt1*^{KK108653}, *Vps33B*^{GD14789} , *Cad99C*^{GD153}, *Hsp83*^{KK101256}, *Duox*^{GD844}, *didum*^{GD1848}, *CG7433*^{GD12238}, *grk*^{KK105496}, *mbc*^{GD6965}, *Ca-alpha1D*^{GD1737}, *Pka-R2*^{KK109446}, *Pkc53E*^{GD11984}, *CAH1*^{KK108727}. For the randomly selected genes, the following VDRC RNAi alleles were used: *Rho-Kinase*^{KK107802}, *Spock*^{GD15076}, *Spatzle*^{KK112908}, *l(1)G0148*^{GD13811}, *SoxN*^{GD4415}, *Rab3*^{KK108633}, *Pka−C2*^{GD4649}, *polo*^{GD7563}, *Cdk7*^{GD4167}, *Pkn*^{KK101337}, *hppy*^{GD12129}, *LIMK1*^{GD9586}, *CycJ*^{GD6936}, *dRSK*^{KK109199}, *cdi*^{GD8731}, *Ddr*^{GD13382}, *Sln*^{KK104306}.

Diazepam treatment

Diazepam was dissolved in 100% ethanol to prepare a stock solution. Diazepam stock solution was diluted in 5% sucrose and 5% yeast-extract solution to prepare solutions corresponding to dosages of 2.5–10 mM. Overnight starved flies were fed with diazepam solutions for ~6-8 h using capillary feeders (CAFE) [S6] at 25°C prior to behavioral testing. Red dye in the food was used to monitor drugged food consumption in the flies. Non-feeding flies were excluded from the analysis. Control flies were fed with carrier solutions mixed with an equivalent amount of ethanol. An identical quantity of ethanol was used across diazepam concentrations, i.e. ethanol was held constant.

Stressors

C-S male flies were used in all stress paradigms. Heat shock was induced by heating the flies to 37°C while in the arena. For restraint stress, each individual fly was immobilized in a small space formed between two nested 10 µl pipette tips (Eppendorf). This arrangement allowed ventilation through the tips but prevented most movement. Flies were restrained for a period of one hour prior to behavioral testing. For single housing, C-S pupae were removed from vials and placed into vials either singly or in groups. Once eclosed, individual males flies were retrieved and transferred to either a second isolation vial or a vial with 24 other males for ten days prior to the assay.

Behavioral assays

For each behavioral assay, batches of 20–25 male flies were isolated under carbon dioxide anesthesia and placed in food vials 2–3 days prior to behavioral recording. Just prior to assay, a batch was knocked into an ice-chilled vial for 20–30 seconds (cold anesthesia) before they were placed individually into an arena with forceps. The wall-following arena was a 1 cm square with a height of 1.6 mm; twenty arenas were laser cut from a sheet of black acrylic to allow simultaneous video analysis of multiple flies. The light/dark box was a 40 × 4 mm rectangular arena; ten such arenas were cut from an acrylic sheet. Each arena was equally divided into two 20 × 4 mm illuminated and dimmed light arenas using transparent plastic sheet on one side and transparent green filter on the

other side. The individual array was covered with a transparent, anti-reflection coated glass sheet (Edmund optics) and was placed in an incubator. The open field was lit from the sides and the light/dark box from below by white LED microscope lamps (Falcon Illumination). To image the flies, an AVT Guppy F-046B CCD camera (Stemmer Imaging) equipped with a 12 mm CCTV-type lens was positioned above the behavioral arenas and connected to a computer via a IEEE 1394 cable. Flies were allowed to freely explore the arena during a 10-minute test session. Recordings were taken for 10 minutes in all assays following similar protocols in rodent and fly [S7-10]. Flies' positions were determined from a live video feed with CRITTA, custom software written in LabVIEW (National Instruments), which extracted centroid x-y position data and recording it to a binary file for offline analysis in MATLAB. For almost all experiments, samples sizes were approximately N = 60, 60, so as to yield a statistical power of ~0.75 when alpha = 0.05, assuming a moderate effect size of 0.5 standard deviations. All sample sizes were precisely N = 60, 60, unless otherwise mentioned. Behavioral tests of control and intervention flies were run in alternation on the same day.

Quantitative PCR

To measure mRNA levels in transgenic animals, batches of 2–3-day-old male flies were isolated from the crosses of *nSyb-Gal4*; *tub-Gal80ts/TM3* with the responder transgene (*i.e., UAS-RNAi* or *UAS-cDNA)* and were either maintained at 18°C or treated for 16–18 h overnight interval(s) at 31°C. For each gene expression analysis in response to stress, stressors were applied to 2–3-day-old C-S male flies. Heads of flies were separated using copper sieves, liquid nitrogen, and dry ice. Five independent total RNA samples were isolated immediately from different groups of control and stressors from ~20 heads for each sample using TriZol (Sigma), and stored at -80°C till further use. The RNA concentration was assessed using NanoDrop. From each condition, 2 µg of RNA was reverse transcribed into cDNA using a cDNA synthesis kit (Applied Biosystems). The expression of each gene was assayed in 96-well optical plates in a 7700 Sequence Detection System (Applied Biosystems) with default settings. All qPCR reactions were performed in duplicate and threshold cycle values (C_t) were averaged. TaqMan-based gene-specific primers were used to efficiently probe the respective gene transcripts. Normalized relative expression levels were calculated with *Rpl32* levels as the internal control. The relative quantification in gene expression was determined using the $2^{-\Delta\Delta Ct}$ method [S11].

Data analysis and statistics

Custom MATLAB scripts were used to analyze behavior and expression data. Fly centroid x-y location data were filtered to exclude unmoving, dead, or missing flies. The filtered dataset was used to calculate the distance from the arena's center in the WAFO assay and percent time spent in the shade in the light/dark choice assay. In the WAFO assay, we used mean distance from the center as

our raw measure of WAFO; this metric was preferred over the 'proportion of time spent in central zone' metric widely used in rodent studies as the latter relies on an arbitrarily defined central area and in our hands had a highly non-normal distribution. Contrasts were reported as standardized mean differences (Hedges' *g*). Hedges' *g* and its bootstrapped 95% confidence interval for the difference between control and experimental animals were calculated with the Measurements of Effect Size toolbox (MES) in MATLAB [S12]. Hedges' *g* is a standardized effect size expressed in units of standard deviation [S13]; specifically, *g* is the preferred variant of Cohen's d that uses SD_{pooled} and adjusts for bias [S14]. Standardized effect sizes have the benefit of allowing combinations across and comparisons between different experimental systems. Because behavioral assay metrics may have a non-linear and/or indirect relationship to internal brain state, standardizing the contrast effect size with the standard deviation has the additional benefit of recasting the data in terms of the controls' behavior, a superior reference point than the chamber size. Standardized effect sizes including Hedges' *g* are conventionally classified as 'trivial' (< 0.2 standard deviation), 'small' (< 0.5), 'medium' (< 0.8) and 'large' (> 0.8) {Borenstein:2011um). The total distance traveled and experimental duration (10 min) were used to calculate the average speed of the fly. Means and their 95% confidence intervals are reported in the format: 'mean [95CI lower bound, upper bound]'. Linear regression was done with the LinearModel.fit function in Matlab. For comparisons of two independent groups, confidence intervals of the primary data and the contrasts were calculated by the bias-corrected and accelerated (BCa) bootstrap method, and P was computed with the Mann-Whitney-Wilcoxon *U* method (using the bootci and ranksum functions respectively). All error bars in all data figures are 95% confidence intervals.

SUPPLEMENTAL REFERENCES

S1. Park, S. K., George, R., Cai, Y., Chang, H. Y., Krantz, D. E., Friggi-Grelin, F., Birman, S., and Hirsh, J. (2006). Cell-type-specific limitation on in vivo serotonin storage following ectopic expression of the Drosophila serotonin transporter, dSERT. J Neurobiol *66*, 452–462.

S2. Yuan, Q., Lin, F., Zheng, X., and Sehgal, A. (2005). Serotonin modulates circadian entrainment in *Drosophila*. Neuron *47*, 115–127.

S3. Colas, J. F., Launay, J. M., Vonesch, J. L., Hickel, P., and Maroteaux, L. (1999). Serotonin synchronises convergent extension of ectoderm with morphogenetic gastrulation movements in Drosophila. Mech Dev *87*, 77–91.

S4. McGuire, S. E., Le, P. T., Osborn, A. J., Matsumoto, K., and Davis, R. L. (2003). Spatiotemporal rescue of memory dysfunction in *Drosophila*. Science *302*, 1765–1768.

S5. Dietzl, G., Chen, D., Schnorrer, F., Su, K.-C., Barinova, Y., Fellner, M., Gasser, B., Kinsey, K., Oppel, S., Scheiblauer, S., et al. (2007). A genome-wide transgenic RNAi library for conditional gene inactivation in Drosophila. Nature *448*, 151–156.

S6. Ja, W. W., Carvalho, G. B., Mak, E. M., la Rosa, de, N. N., Fang, A. Y., Liong, J. C., Brummel, T., and Benzer, S. (2007). Prandiology of Drosophila and the CAFE assay. Proc Natl Acad Sci USA *104*, 8253–8256.

S7. Parks, C. L., Robinson, P. S., Sibille, E., Shenk, T., and Toth, M. (1998). Increased anxiety of mice lacking the serotonin1A receptor. Proc Natl Acad Sci USA *95*, 10734–10739.

S8. Liu, L., Davis, R. L., and Roman, G. (2007). Exploratory activity in *Drosophila* requires the kurtz nonvisual arrestin. Genetics *175*, 1197–1212.

S9. Gross, C., Zhuang, X., Stark, K., Ramboz, S., Oosting, R., Kirby, L., Santarelli, L., Beck, S., and Hen, R. (2002). Serotonin1A receptor acts during development to establish normal anxiety-like behaviour in the adult. Nature *416*, 396–400.

S10. Martin, J., Ernst, R., and Heisenberg, M. (1999). Temporal pattern of locomotor activity in Drosophila melanogaster. Journal of Comparative Physiology A: Sensory.

S11. Livak, K. J., and Schmittgen, T. D. (2001). Analysis of relative gene expression data using real-time quantitative PCR and the 2(-Delta Delta C(T)) Method. Methods *25*, 402–408.

S12. Hentschke, H., and Stüttgen, M. C. (2011). Computation of measures of effect size for neuroscience data sets. Eur J Neurosci *34*, 1887–1894.

S13. Cumming, G. (2012). Understanding the New Statistics: Effect Sizes, Confidence Intervals, and Meta-Analysis.

S14. Borenstein, M., Hedges, L. V., Higgins, J., and Rothstein, H. R. (2011). Introduction to meta-analysis (John Wiley & Sons, Ltd).
